# Integrated 3D light sheet and 2D multiplex imaging for deep histological characterization of somatic mouse glioblastoma

**DOI:** 10.1016/j.isci.2026.117476

**Published:** 2026-09-08

**Authors:** Marie Catherine Tiveron, Nathalie Coré, Kevin Bigott, Yliana Huriaux Fontana, Maria Caccavale, Léna Vilvandre, Fabio El Yassouri, Victoria Schoppel, Silvia Rüberg, Melanie Jungblut, Dominique Figarella-Branger, Aurélie Tchoghandjian, Andreas Bosio, Harold Cremer

**Affiliations:** 1 Aix-Marseille University, CNRS, IBDM, Marseille, France; 2 Miltenyi Biotec B.V. & Co. KG, Bergisch Gladbach, Germany; 3 Aix-Marseille University, CNRS, INP, Institute Neurophysiopathol, GlioME Team, Marseille, France; 4 Aix-Marseille University, Réseau Préclinique et Translationnel de Recherche en Neuro-Oncologie, Plateforme PETRA”TECH”, Marseille, France

**Keywords:** somatic glioblastoma model, multiplex imaging, light sheet imaging, neural stem cells, brain electroporation

## Abstract

Glioblastoma is a devastating brain cancer for which patient survival has remained largely unchanged for decades, underscoring the need for improved disease modeling and analytical tools. Neural stem cells have been identified as cells of origin of glioblastoma, leading to the development of somatic lineage models. Such models have been deeply characterized by sequencing but systematic histological analyses remain limited. Here, we present a multimodal histological characterization of a somatic glioblastoma mouse model. Using 3D light-sheet imaging, we show that the model is highly reproducible and enables quantitative assessment of tumor growth across cohorts. Through multiplex imaging with MACSima™ Imaging Cyclic Staining, we map the cellular landscape and molecular architecture of the tumors and their environments, and provide a curated resource of mouse-compatible antibodies. Finally, we demonstrate that tissue clearing and light-sheet microscopy can be seamlessly combined with multiplex imaging, enabling spatial proteomic characterization of a 3D pre-defined tumor.

## Highlights

• 3D analyses of tumor growth in space and time in a somatic mouse model for glioblastoma
• A spatial proteomic map of tumor and microenvironmental cell states in mice
• Integration of tissue clearing, 3D microscopy and 2D multiplex imaging in one workflow

## Graphical abstract

**Figure:**
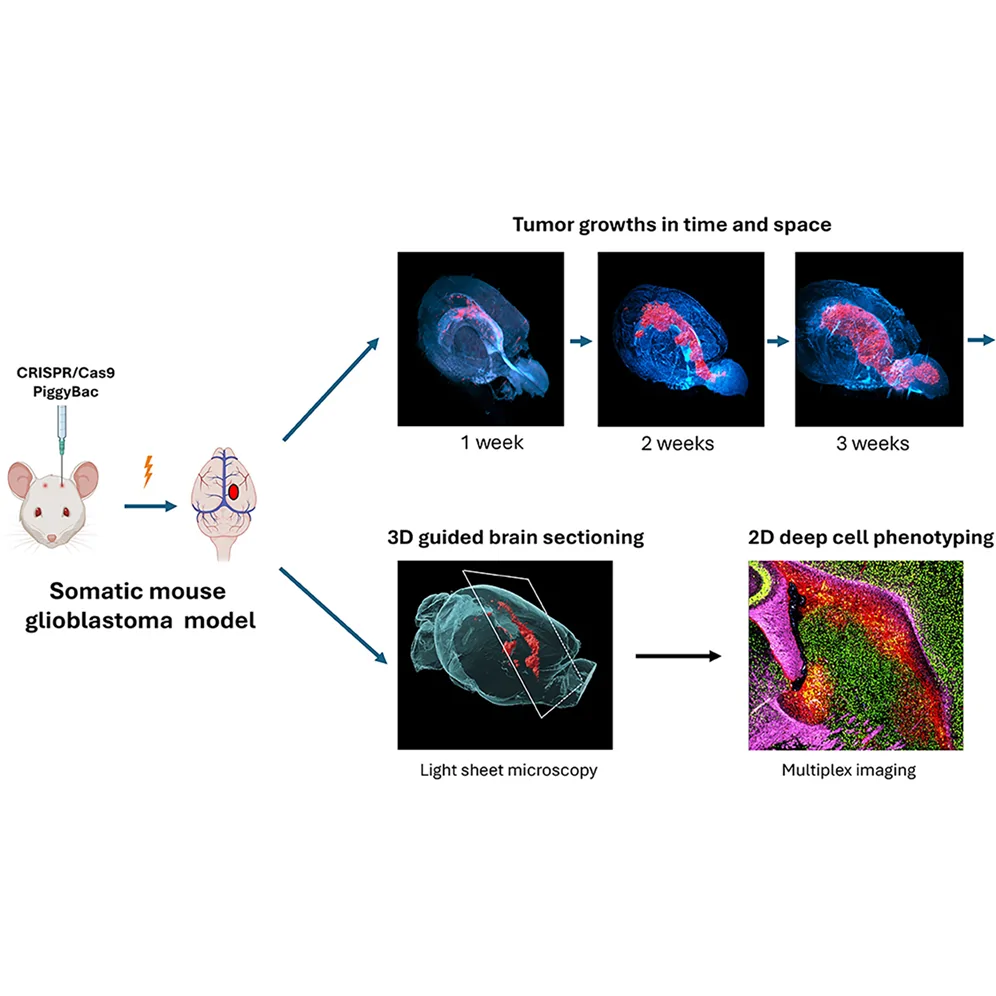

## Teaser

Biological sciences; Neuroscience; Cancer; Methodology in biological sciences

## Introduction

Glioblastoma (GBM) is the most common malignant brain tumor in humans, yet effective therapies remain unavailable and median survival still falls below 20 months.^1^^,^^2^ Despite decades of research, clinical progress has been limited, highlighting the need for experimental systems that more precisely model the biology of the disease, in concert with the development of advanced analytic tools for their study.

The identification of subventricular zone (SVZ) neural stem cells (NSCs) as cells of origin for GBM^3^ provided an entry point. Indeed, *in vivo* electroporation enables efficient genetic manipulation of NSCs localized in different regions of the ventricular wall,^4^^,^^5^^,^^6^ and CRISPR-Cas9–based somatic lineage models have been used to generate high-grade IDH1-wildtype gliomas in fully immunocompetent mice.^3^^,^^7^^,^^8^^,^^9^^,^^10^ These somatic NSC-based models offer several analytical advantages. They begin from a defined cell population, allow fluorescent lineage tracing of cancer cells within the tumor microenvironment and the wider brain parenchyma, and preserve intact immune interactions. They have already been used to dissect, for example, transcriptional regulators of tumor initiation,^9^ the biology of the invasive margin,^8^ mechanisms of brain infiltration,^11^ and the immune landscape of GBM.^12^^,^^13^^,^^14^

Thus, somatic mouse models are of increasing importance for GBM research, highlighting the need for efficient and reproducible approaches for their analysis. Bulk and single-cell sequencing have been widely used in this context and provided valuable insights into cell populations and molecular regulation. However, deep histological analyses of tumor development, from earliest stages to complex three-dimensional tumors spanning widely through the entire brain, are currently scarce. Also, systematic insight into the proteomic landscape of such tumors and their surrounding micro- and macroenvironments is limited, which is in part due to the scarcity of well-validated, mouse-specific antibodies.

Here, we present an in-depth histological and spatial analytical framework for the study of somatic GBM mouse models. Using brain electroporation in concert with CRISPR/Cas9 technology and PiggyBac recombination, we established a tumor induction system that recapitulates the classical histopathological features of GBM and show that it allows studying tumorigenesis from early stages post-transformation. Using tissue clearing and 3D light-sheet fluorescence microscopy (LSFM), we tracked tumor development and demonstrated that the model is rapid, highly reproducible, and quantitatively measurable at the whole-brain scale. We then applied automated cyclic immunofluorescence staining (MICS, MACSima™ Imaging Cyclic Staining)^15^ to generate a comprehensive spatial proteomic map of tumor and microenvironmental cell states, providing a curated resource of functional, mouse-compatible antibodies for GBM research. Finally, we show that LSFM-based 3D mapping can be seamlessly integrated with subsequent MICS analysis, enabling molecular profiling of pre-identified tumor regions with highest spatial precision.

## Results

### GBM induction by electroporation

**Figure 1:**
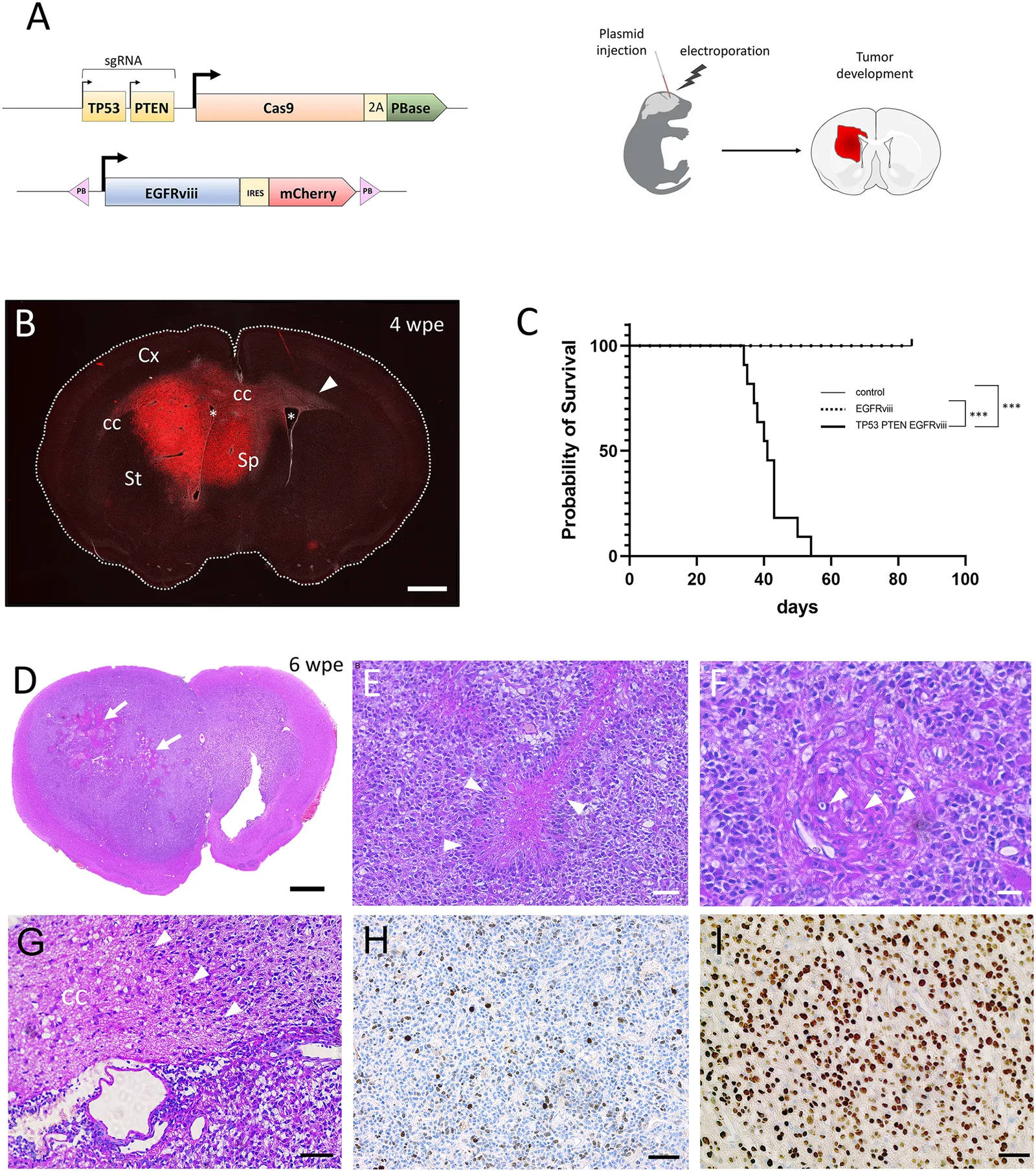
Induction of GBM using plasmid electroporation (A) Schematic representation of the plasmids used in electroporation. One episomal plasmid carries the CRISPR/Cas9 system mediating the inactivation of *TP53 and PTEN* and supports PiggyBac transposase expression that allows genomic integration of a second plasmid encoding *EGFRvIII* and *nuclear mCherry (mCherry)* genes. U6 promoters drive the expression of the guide RNAs, while *Cas9* and *hyPBase* are jointly transcribed under the control of Cbh promoter. *EGFRvIII* and *nls-mCherry* are expressed from a single transcription unit, also driven by Cbh promoter. After electroporation, only cells targeted with both plasmids will induce tumors that permanently express mCherry (small arrows: U6 promoter; large arrows: Cbh promoter); sgRNA: guide RNA; hyPBase: Hyper PiggyBac transposase; PB: piggyBac elements; P2A: P2A auto-cleaving peptide; IRES: Internal Ribosome Entry Site. (B) Brain coronal section at 4 weeks post electroporation (4wpe). Stars indicate lateral ventricles; arrow head shows tumor cells invading the contralateral brain hemisphere via the corpus callosum. (C) Kaplan-Meier survival analyses of control (*n* = 10), EGFRvIII (*n* = 12) and TP53/PTEN/EGFRvIII (*n* = 11) mice. ∗∗∗: *p* < 0.00001. (D–I) Histological analyses on a brain coronal section at 6 wpe. (D–G) Hematoxylin-Eosin staining with high magnification shows necrotic palisade highlighted by arrow heads (E), glomerular vessels with mitotic figures (arrowheads) in endothelial cell (F) and tumor cells invading the corpus callosum (G, arrowheads highlight the migration front). (H and I) Immunohistochemistry for Ki-67 (H) and Olig2 (I). cc: corpus callosum; Cx: cortex; Sp: septum; St: striatum. Scale bars, 1000 μm (B and D); 50 μm (E, G, H, and I); 20 μm (F).

We induced brain tumors using an electroporation-based two-vector system (Figure 1A). This approach simultaneously inactivates two tumor suppressor genes, *TP53* and *PTEN,* and constitutively activates the Epidermal growth factor receptor (EGFR) pathway via EGFRvIII overexpression—a mutation profile associated with the classical subtype of GBM.^16^ Specifically, *TP53/PTEN* gene inactivation is driven by the CRISPR/Cas9 system,^3^ while the PiggyBac transposase (PBase) system^17^ mediates genomic integration of a second plasmid that ensures high-level expression of EGFRvIII and mCherry in all targeted cells and their lineages.

Co-electroporation of both plasmids into neural stem cells (NSCs) lining the forebrain ventricles resulted four weeks later in the formation of large mCherry-positive tumors in all animals. These tumors extended into adjacent ipsilateral structures, including the striatum (St), corpus callosum (CC), and septum (Sp; Figure 1B). In most brains, at later stages, fluorescent cells were also detected in the contralateral hemisphere, with the CC appearing as the primary infiltration route (arrowhead).

Survival analysis revealed that animals receiving both plasmids began to show morbidity and mortality at approximately 5 weeks post-electroporation (wpe), with progression over the following three weeks (Figure 1C). In contrast, animals receiving only a control plasmid, or transfected with the EGFRvIII-expressing plasmid and PBase alone, exhibited no lethality during the observed period (Figure 1C).

To characterize the induced tumors, we first employed classical histopathology. Representative sections from electroporated animals at 6 wpe—when tumors were advanced—revealed extensive infiltration of both hemispheres and large tissue lesions (Figure 1D, arrows). At higher magnification, necrotic palisades (Figure 1E, arrowheads) and glomerular vessels with endothelial-capillary proliferation, a hallmark of angiogenesis, were evident throughout the tumor (Figure 1F; mitoses indicated by white arrowheads). In several animals, a distinct invasion front was visible within the corpus callosum (Figure 1G, arrowheads), consistent with previous findings that white matter tracts serve as primary infiltration routes for cancer cells.^18^^,^^19^ Immunohistochemistry for the proliferation marker Ki-67 and the glial marker Olig2 showed a high density of positive cells within the tumors (Figures 1H and 1I). Thus, the two-vector lineage model efficiently induced tumors that recapitulate the major histopathological hallmarks of stage four GBM.^20^

### Imaging the earliest stages of tumor development

As *in vivo* electroporation targeting NSC along the ventricular wall^4^ provides a precise starting point and a defined population of cells of origin, we studied the earliest events after transformation. The GBM induction system was electroporated into the lateral (LWE) or dorsal (DWE) ventricular walls, as previously described.^5^^,^^21^^,^^22^ For these experiments, cytoplasmic GFP was introduced as the fluorescent marker, enabling detailed analysis of cell size and morphology.

**Figure 2:**
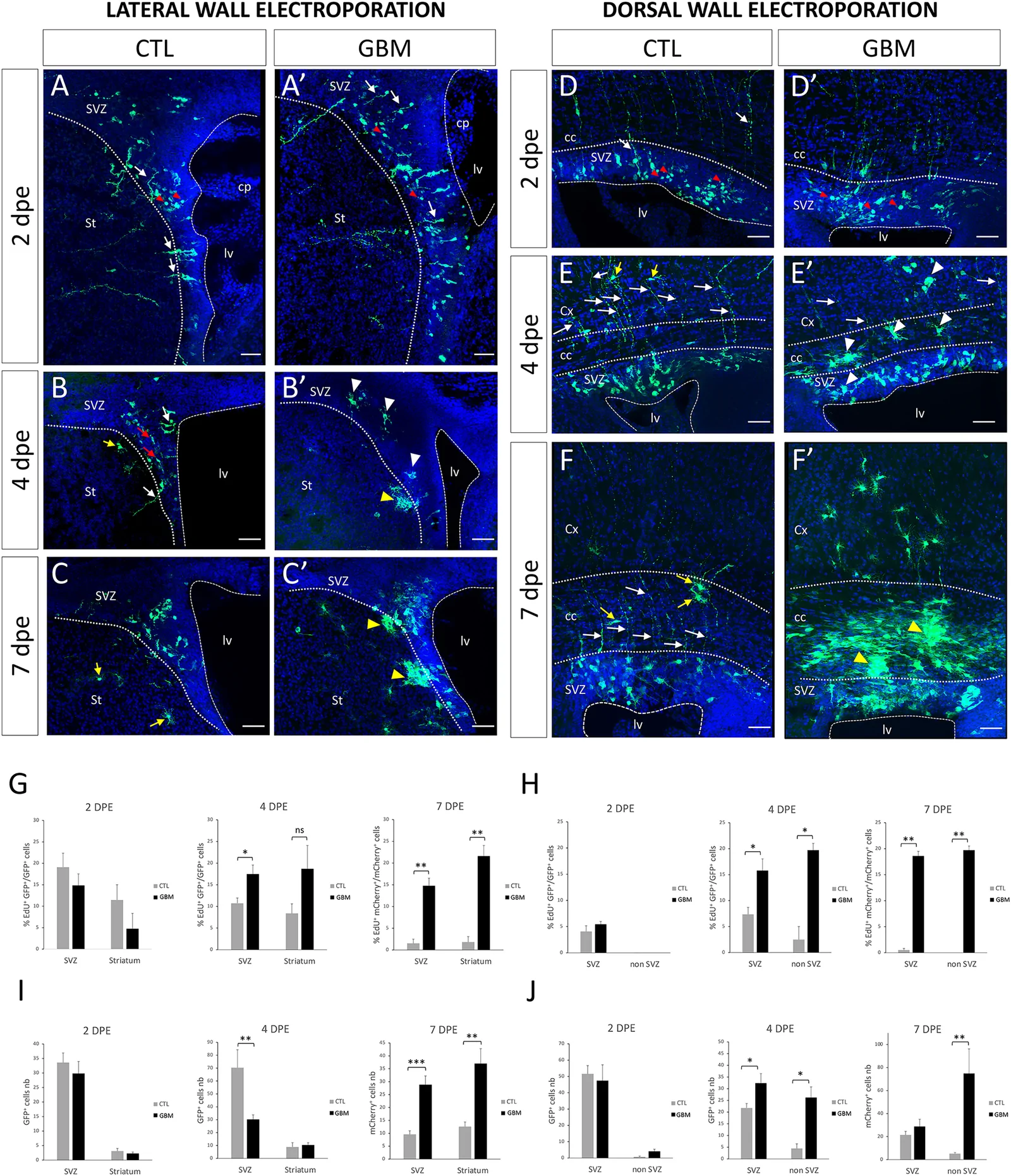
Characterization of early cellular events following GBM induction Representative coronal brain sections showing GFP-positive cells and their progeny after lateral (A–C and A′–C′) or dorsal (D–F and D′–F′) wall electroporation with control (A–F) or GBM inducing (A′–F′) plasmids. Animals were sacrificed at 2 dpe (A,A′, D,D′), 4 dpe (B,B′,E,E′), or 7 dpe (C,C′,F,F′). White arrows indicate the process of Radial Glia progenitors, red arrows show migrating neuroblasts, red arrow heads show presumable neuronal progenitors in the SVZ, yellow arrows point to cells in the striatum and cortex harboring multi-polar morphology resembling the expected glial cells, white arrow heads highlight abnormal multipolar cells, yellow arrow heads show cell aggregates never observed in controls. Quantification of proliferation rate by EdU incorporation in targeted cells after LWE (G) or DWE (H) in control or GBM condition. Quantification of targeted cell numbers after LWE (I) or DWE (J). Control: LWE *n* = 11, DWE *n* = 10, GBM: LWE *n* = 15 (n represents the total number of mice in 2 independent experiments) DWE *n* = 5 mice in one experiment. Histograms represent mean values ±SEM. Statistical test (Mann-Whitney U test): ∗*p* ≤ 0.05, ∗∗*p* ≤ 0.01, and ∗∗∗*p* ≤ 0.001. Scale bars, 50 μm. cc: corpus callosum, cp: choroid plexus, Cx: cortex, lv, lateral ventricle, St: striatum, SVZ: subventricular zone.

Under control conditions (Figures 2A–2F), the expected progression of postnatal neurogenesis was observed following both LWE and DWE. At 2 dpe, GFP-positive neural stem cells (NSCs) with radial glia morphology were present in the SVZ (Figure 2A), consistent with previous observations that postnatal electroporation specifically targets this cell type.^4^ Additionally, individual cells lacking radial processes were detected, likely representing proliferating transit-amplifying progenitors derived from the initially targeted NSCs (Figures 2A–2D, red arrowheads), which generate migratory precursors destined for the OB.

At 4 dpe, the lateral SVZ still contained a small number of radial glia-like cells (Figure 2B, white arrows), while cells with the bipolar morphology and tangential orientation characteristic of migrating OB neuroblasts were observed (Figure 2B, red arrows). Occasional GFP-positive cells in the adjacent striatum likely represented SVZ-derived glial precursors (Figure 2B, yellow arrow).^23^ In the dorsal SVZ, numerous GFP-positive radial glia-type cells were present (Figure 2E, white arrows), while elongated morphologies indicating the presence of neuronal precursors were rare, consistent with the lower neurogenic output of this compartment.^24^ GFP-positive cells in the overlying cortex likely corresponded to SVZ-derived glial precursors (Figure 2E, yellow arrows).

By 7 dpe, both the lateral (Figure 2C) and dorsal (Figure 2F) SVZ still contained high numbers of GFP-positive cells. Some of these exhibited migratory morphologies, consistent with neuroblast translocation toward the OB. Scattered multipolar GFP-positive cells in the striatum and corpus callosum likely represented newly generated glial cells (Figures 2C–2F; yellow arrows).

We next examined the effects of LWE and DWE under GBM-inducing conditions (Figures 2A′–2F′). At 2 dpe, GFP-positive cells were almost exclusively localized in the SVZ, with only a few radially oriented cells positioned in the overlying CC. Cell morphology, positioning, and numbers (Figures 2A′, 2D′, 2I, and 2J), as well as proliferation rates (Figures 2G and 2H), were comparable to controls.

However, by 4 dpe, transformed cells exhibited markedly altered distribution. The percentage of proliferating GFP-positive cells increased significantly in both the lateral and dorsal SVZ (Figures 2G and 2H), resulting in higher total GFP-positive cell numbers along the dorsal wall (Figure 2J). Notably, this increase was not detected in the lateral wall, likely due to the rapid succession of neurogenic divisions occurring here between 2 and 4 dpe under normal conditions (Figure 2I).

Morphologically, transformed GFP-positive cells in the lateral SVZ no longer exhibited the bipolar orientation typical of radial glia or migratory precursors. Instead, they displayed multiple processes (Figure 2B′, white arrowheads) and tended to form clusters, accumulating at the striatal border (Figure 2B′, yellow arrowhead). In the dorsal SVZ at 4 dpe, the loss of radial glia morphology, acquisition of multiprocess phenotypes, and cluster formation were even more pronounced (Figure 2E′, white arrowheads).

By 7 dpe, these abnormalities were dramatically amplified. Large aggregates of transformed cells accumulated along both the lateral and dorsal ventricular walls (Figures 2C′–2F′, yellow arrowheads), proliferation was strongly increased (Figures 2G and 2H), and multipolar cells invaded the neighboring striatum after LWE, as well as the corpus callosum and cortex after DWE (Figures 2I and 2J).

In conclusion, the introduction of transforming driver mutations into SVZ NSCs rapidly disrupted normal neurogenesis, inducing profound abnormalities—including increased proliferation, altered morphology, and accumulation of transformed cells adjacent to the targeted progenitor zones. These early events mark the onset of tumor initiation in this model.

### Tumor growth in space and time

To systematically describe and quantify GBM growth in its integrity, we combined tissue clearing with LSFM, enabling 3D analysis of tumor size and shape in whole brains. Mice were electroporated via LWE, DWE, or medial wall electroporation (MWE) with either control plasmids or the GBM-inducing system. For tissue clearing, we used the CUBIC protocol,^25^ which preserves the fluorescence of marker proteins such as mCherry.

**Figure 3:**
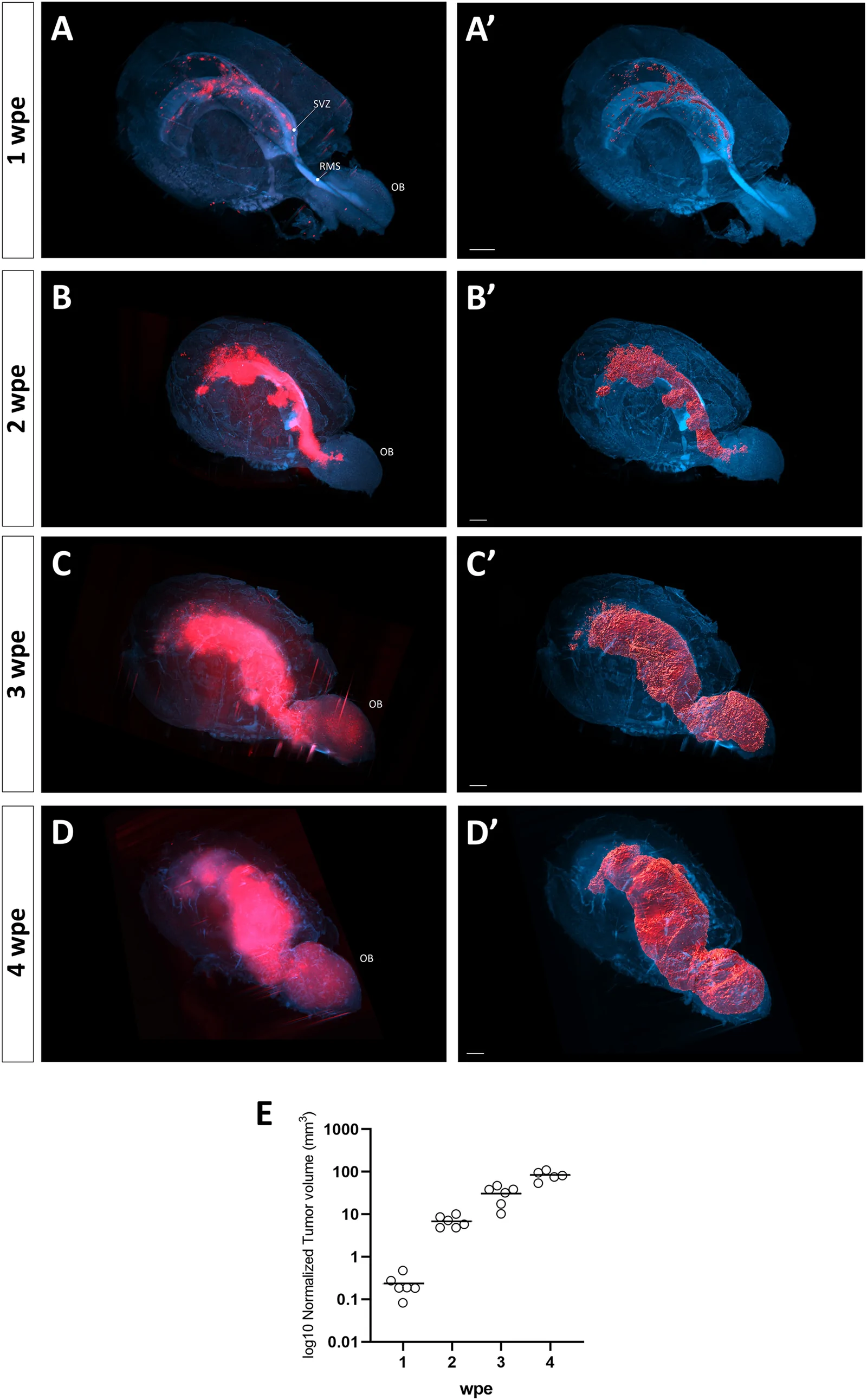
Visualization of tumor progression by 3D imaging (A–D) 3D views after LSFM imaging of mCherry-positive tumors in hemibrains at 1, 2, 3 and 4 wpe after targeting the lateral wall of the lateral ventricle (LWE). Nuclei staining with TOPRO3 (blue) allows positioning of the tumor in the brain. Note the cell-dense SVZ lining the brain ventricles and the rostral migratory stream representing the rostral extension of the SVZ into the OB. (A′–D′) Corresponding 3D images after segmentation of the mCherry signal showing the tumor surface. 3D animations are presented under Videos S1, S2, S3, and S4. (E) Growth of tumor volume at 1, 2, 3, and 4 wpe in animals after LWE. Each circle represents one animal. The mean volume per time point per condition is drawn as a bar. Scale bars, 1000 μm.

One week post-electroporation (wpe), small mCherry-positive clusters appeared along the ventricular wall (Figures 3A, and 3A′; Video S1), consistent with individually targeted neural stem cells (NSCs) as the cells of origin.^4^^,^^26^ By 2 wpe, individual clusters merged into large mCherry-positive masses covering the ventricular wall and extending into neighboring structures, indicating the fusion of clones into a common tumor (Figures 3B and 3B′; Video S2). Over the next two weeks, tumors continued to expand, invading surrounding tissues such as the olfactory bulb (OB) and cortex (Cx, Figures 3C, 3C′, 3D, and 3D′; Videos S3 and S4). Tumors induced by DWE or MWE exhibited similar growth and invasion patterns over time (Figures S1A–S1D).

Video S1. Representative 3D projection of experimental GBM at 1wpe

Video S2. Representative 3D projection of experimental GBM at 2wpe

Video S3. Representative 3D projection of experimental GBM at 3wpe

Video S4. Representative 3D projection of experimental GBM at 4wpe

Quantification of tumor size demonstrated highly reproducible growth across animals during the analyzed period (Figures 3E, S1C, and S1D). Notably, tumor development—and consequent lethality (Figure 1C)—was rapid compared to other electroporation-based models.^8^^,^^9^^,^^10^ This rapid progression is likely due to the high expression levels of EGFRvIII driven by the Cbh promoter, as well as the genomic integration of multiple copies via the PiggyBac system.

### Multiplex imaging analyses by MICS

To provide deep insight into the histological cellular landscape and molecular composition of the induced tumors, as well as their micro- and macro-environments, we used ultra-high-content imaging based on MICS technology,^15^ studying tumors induced in the different ventricular walls at 2 wpe, when compact GBM were observed with full penetrance (Figures 3B, S1A, and S1B).

Initially, a total of 77 fluorochrome-conjugated antibodies, most of these validated for human tissues but not in mice, were tested on 5 different sections prepared from brains containing tumors induced by either LWE, DWE or MWE. Of these, 38 markers showed high-quality staining at the cellular level and were analyzed in depths in subsequent steps (Table 1, individual images Figure S2).

**Table 1:** Antibodies panel for MICS

Antigen	Clone	Dilution	Order no.	Fluorochrome	Company
ACSA-2	REA969	50	130-116-243	FITC	Miltenyi Biotec
AN2	REA989	50	130-116-376	PE	Miltenyi Biotec
CD11b	M1/70.15.11.5	50	130-113-235	PE	Miltenyi Biotec
CD11c	REA754	50	130-110-837	FITC	Miltenyi Biotec
CD18	REA1226	50	130-124-120	APC	Miltenyi Biotec
CD29	REA1074	50	130-119-165	PE	Miltenyi Biotec
CD34	REA383	50	130-117-775	FITC	Miltenyi Biotec
CD38	REA616	50	130-122-955	FITC	Miltenyi Biotec
CD44	REA664	50	130-119-121	APC	Miltenyi Biotec
CD45	REA737	50	130-110-796	FITC	Miltenyi Biotec
CD49e	REA1183	50	130-122-072	PE	Miltenyi Biotec
CD54 (ICAM-1)	REAL289	50	130-115-986	APC	Miltenyi Biotec
CD105	REA1058	50	130-118-174	PE	Miltenyi Biotec
CD106 (VCAM-1)	REA971	50	130-116-322	FITC	Miltenyi Biotec
CD146 (LSEC)	REA1064	50	130-118-406	FITC	Miltenyi Biotec
CD169 (Siglec-1)	REA197	50	130-125-523	PE	Miltenyi Biotec
CD171 (L1CAM)	555	10	130-102-221	APC	Miltenyi Biotec
EGF Receptor	REA939	50	130-115-506	APC	Miltenyi Biotec
EphA2	REA579	10	130-109-140	APC	Miltenyi Biotec
GFAP	REA335	50	130-118-351	PE	Miltenyi Biotec
GLAST (ACSA-1)	ACSA-1	50	130-118-344	PE	Miltenyi Biotec
Integrin α7	3C12	50	130-120-812	PE	Miltenyi Biotec
Ki-67	REA183	50	130-120-417	PE	Miltenyi Biotec
NeuN	REA1131	50	130-119-492	FITC	Miltenyi Biotec
MBP	REA1154	50	130-120-342	PE	Miltenyi Biotec
Mer	REA477	50	130-128-215	APC	Miltenyi Biotec
MHC Class II	REA813	50	130-112-386	FITC	Miltenyi Biotec
Mog	REAL797	50	130-128-468	PE	Miltenyi Biotec
Nestin	REA575	50	130-120-164	APC	Miltenyi Biotec
Neurofilament	REA1127	50	130-119-496	PE	Miltenyi Biotec
O4	REA576	50	130-119-897	APC	Miltenyi Biotec
P2X7R	REA875	50	130-114-328	FITC	Miltenyi Biotec
Phosphotyrosine	REA374	11	130-105-448	APC	Miltenyi Biotec
Plexin-B2	REA445	10	130-106-335	PE	Miltenyi Biotec
Prominin-1	MB9-3G8	50	130-117-784	PE	Miltenyi Biotec
SSEA-1	REA321	50	130-117-689	PE	Miltenyi Biotec
Synapsin-1	REA1125	50	130-119-358	FITC	Miltenyi Biotec
Tyrosine Hydroxylase	REA1159	50	130-120-350	FITC	Miltenyi Biotec

**Figure 4:**
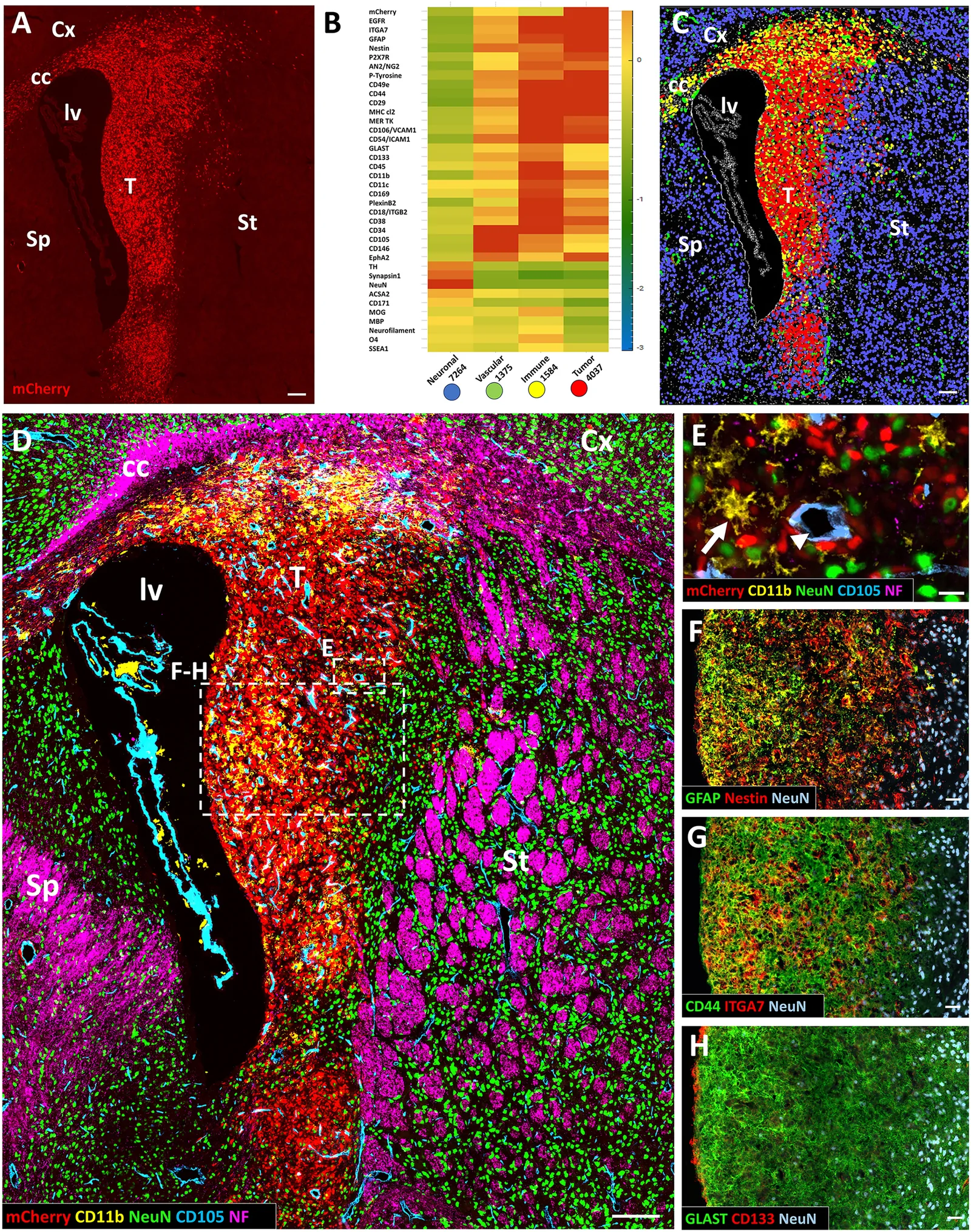
High-resolution histological analyses using MICS (A) Coronal section used for MICS analyses showing a tumor (T) obtained from LWE at 2wpe. Nuclear mCherry allows identification of cancer cells. (B) Heatmap presentation of protein expression in segmented cells. Clusters were defined by initial gating of neurons (NeuN), Vascular cells (CD105), myeloid cells (CD11b), and mCherry (cancer cells). Number of cells in each cluster is indicated. (C) Spatial representation of the four cell clusters as defined in B. (D–H) Ultrahigh-content imaging allows a detailed histological overview of the tumor and its microenvironment. (D) Overview image of the tumor and its environment showing selected markers used for clustering in B (NeuN, CD105, CD11b, mCherry). Neurofilament (NF) was added to highlight axon tracts). Boxed areas are presented at high magnification in (E–H). (E) Example of high-resolution histology obtained with MICS showing CD11b^+^ myeloid cells (arrow) and a CD105-labeled blood vessel (arrowhead) surrounded by mCherry^+^ cancer cells. (F–H) Classical tumor markers (GFAP, Nestin, CD44, ITGA7, GLAST, CD133) show differential labeling in the tumor. Scale bars, 100 μm (A, C, and D), 20 μm (F–H), and 5 μm (E). cc: corpus callosum; Cx: cortex; lv: lateral ventricle; Sp: septum; St: striatum.

After segmentation and *Z* score normalization of a coronal section from an LWE-induced tumor at 2 wpe (Figure 4A), we used the MACSiQ software (Miltenyi Biotec) to define four major cell populations based on the expression of canonical markers: neurons (NeuN^+^, *N* = 7,264), endothelial cells (CD105/endoglin^+^, *N* = 1,375), myeloid cells (CD11b^+^, *N* = 1,584), and cancer cells (mCherry^+^, *N* = 4,037). Expression profiling of the four populations (heatmap presentation Figure 4B) confirmed that NeuN^+^ cells co-expressed neuronal markers such as TH, synapsin, and CD171(L1-CAM). CD105^+^ endothelial cells showed the highest levels of vasculature-associated markers, such as CD146, CD34, and EphA2. CD11b^+^ myeloid cells displayed a strong correlation with immune markers such as CD45, CD18, CD11c, and CD169. Finally, mCherry^+^ cancer cells were characterized by robust expression of tumor-associated markers including CD44, nestin, GFAP, and P2X7R.^27^ Overall, marker clustering within each cell type (spatial presentation of clusters in Figure 4C) was coherent, supporting the accuracy of the segmentation and classification pipeline, and further validating the model for the study of brain tumors and their associated microenvironment.

We combined individual markers to provide a detailed histological overview of the tumor, as well as its micro- and macro-environments. We used mCherry for cancer cells (red), CD11b for myeloid cells (yellow), CD105 for vascular endothelial cells (blue), NeuN for neurons (green), and neurofilament for axonal projections (magenta; Figure 4D; high-magnification view of field E in Figure 4E). All main cell types were distinguishable, with a clear separation and complementarity between mCherry-positive cancer cell nuclei and NeuN-positive neuronal nuclei (Figures 4D and 4E). Also, strong accumulation of myeloid cells and large-diameter blood vessels was evident within the tumor area (Figures 4D and 4E). Analysis of coronal sections through tumors induced by DWE and MWE provided images of comparable complexity and quality (Figures S3A–S3D).

Next, we focused on the presence of classical tumor markers. In agreement with the astrocytic origin of the induced tumors from postnatal NSCs,^28^ GFAP was strongly expressed in tumor regions proximal to the lateral ventricle, the site of origin of transformed cells, but weaker in distal regions. In contrast, Nestin, a marker for undifferentiated brain cells and associated with higher-grade gliomas and lower patient survival,^29^^,^^30^ showed strong and homogeneous staining throughout the entire induced tumor, including its invasive margin (Figure 4F).

Comparably, CD44 was widely and homogeneously expressed over the entire tumor area, in line with its constitutive expression on human tumor cell lines and human GBM^31^ (Figure 4G). In contrast, ITGA7 showed strongest expression in defined groups of tumor cells, in agreement with previous findings that identified the integrin as a functional marker for GBM stem cells.^32^

The astrocytic glutamate aspartate transporter GLAST^33^ showed strongest expression in ventricle-proximal regions of the tumor, extending at lower levels into distal areas (Figure 4H), comparable to the expression of GFAP (Figure 4F). Finally, CD133/prominin-1 was strongly present on cells lining the ventricular wall, in agreement with its well-described expression by a subpopulation of ciliated ependymal cells (Figure 4H^34^).

**Figure 5:**
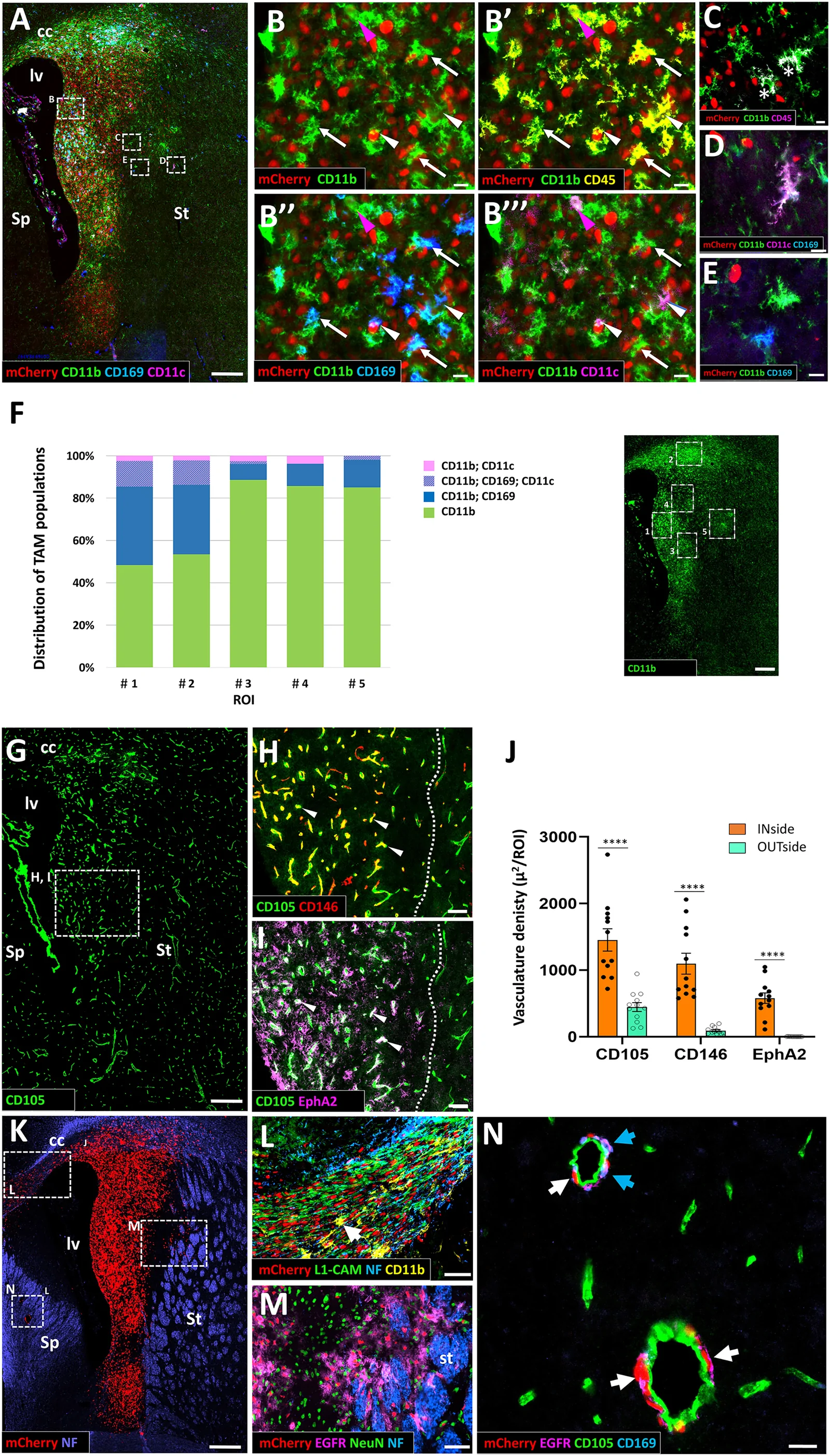
High-resolution histological analyses of GBM hallmarks using MICS The same section as shown in Figure 4 was analyzed in different cellular and molecular contexts using relevant marker combinations. (A–F) Tumor immune microenvironment. (A) Overview of the section labeled by mCherry (cancer cells), CD11b, CD169, and CD11c. Boxed areas delineate regions shown at high magnification in B–E. (B) High magnification of a field of view within the tumor. As expected CD11b-positive cells co-express CD45, a general marker for myeloid cells and a marker of activated myeloid cells (B′). Subpopulations of myeloid cells are identified by the presence of CD169 (B″) and CD11c (B‴) (white arrowheads). Arrows indicate CD11b^+^; CD169^+^ cells that do not express CD11c. Magenta arrowheads show CD11b^+^/CD11c^+^ cells. (C–E) High magnification of fields of view at the periphery of the core tumor, where individual cancer cells invade the brain parenchyma. Myeloid cells are identified by CD11b and CD45 co-labeling in C (white signal; asterisk). (F) Distribution of myeloid cell subpopulations in five different ROIs (shown on the left) within the core tumor (#1,2), the tumor margin (#3,4), and in the surrounding parenchyma comprising scarce cancer cells (#5). (G–J) Tumor-associated angiogenesis. (G) Expression of the pan-endothelial marker CD105 reveals higher vasculature density within the tumor compared to surrounding parenchyma. Boxed area is presented at high magnification in H and I. (H) Compared to CD105, CD146 shows relatively stronger expression in tumor-associated blood vessels than in non-tumoral blood vessels. (I) EphA2 is confined to the tumor and associated with expression on CD105-positive endothelial cells (arrowheads). Diffuse staining surrounding blood vessels represents EphA2 expression by other cells. Dashed lines in H and I represent the tumor boundary. (J) Quantification of vasculature density inside and outside the tumor area, using different vessel markers (*n* = 12 ROIs; see in M&M for ROIs description). (K–N) Tumor cell invasion. (K) General view of the tumor (mCherry) surrounded by fiber tracts identified by neurofilament (NF) staining. Boxed areas delineate fields of view presented at high magnification in L–N. (L and M) Migration along neural fibers. (L) In the corpus callosum, cancer cells are aligned with L1CAM- and NF-positive axonal fibers. Some CD11b-positive myeloid cells are present in the invaded CC (arrow). (M) At the margin of the core tumor, mCherry^+^/EGFR^+^ cancer cells are associated with axon bundles of the striatum identified by NF labeling. (N) Migration along the vasculature. In the septum, individual mCherry^+^/EGFR^+^ cancer cells are in tight association with CD105-expressing vessel endothelial cells (white arrows) and accompanied by CD169-expressing peripheral tumor-associated macrophages (blue arrows). Scale bars, 250 μm (A, F, G, and K), 50 μm (H, I, L, and M), 10 μm (B-B‴,N), 5 μm (C, D, and E). cc: corpus callosum; lv: lateral ventricle; Sp: Septum; St: Striatum. Histograms represent mean value +/- SEM. Statistics: ∗∗∗∗ = *p* value <0.0001 (Mann-Whitney U test).

Next, we examined the tumor immune microenvironment (Figures 5A–5E) and quantitatively assessed the spatial distribution of myeloid subsets in different regions of the tumor and the environment. CD45^+^/CD11b^+^ blood derived and brain-resident microglial cells were highly enriched within the tumor core (Figures 5A, 5B, 5B′, and 5F; compare Figure 4D). Within this compartment, a substantial subset also expressed CD169, consistent with the presence of blood-monocyte-derived antitumoral macrophages infiltrating from the circulation^14^ (Figures 5B″–5F; white arrows). Among these, a minor population additionally expressed CD11c, potentially representing DCs or priming microglia (Figure 5B‴; white arrowhead). In contrast, CD11b^+^/CD11c^+^ double-positive cells (Figures 5B–5B‴; magenta arrowhead),^35^ were detected at very low levels at this tumor stage (Figure 5F).

Beyond the tumor core, myeloid cells were also present at the invasive margin, where individual mCherry^+^ cancer cells infiltrated the surrounding parenchyma (Figures 5C–5E). Here, the presence of CD45^+^/CD11b^+^ cells (Figure 5C) indicated early immune recognition of invading cancer cells. CD11b^+^/CD169^+^ cells were present in lower numbers than in the tumor core, and CD11b^+^/CD169^+^/CD11c^+^ cells were very rare (Figures 5D–5F).

Thus, the tumor immune microenvironment contains a spatially organized mix of resident microglia and infiltrating myeloid cells, with their marker profiles shifting from the tumor core to the invasive margin.

Then we investigated the expression of markers associated with angiogenesis, another hallmark of GBM (Figures 5G–5J). CD105 (Endoglin) was strongly expressed on blood vessels throughout the brain, with significantly higher expression levels in the tumor area versus non-tumor brain tissue, in agreement with the expected high degree of neo-angiogenesis (Figures 5G–5J).^36^^,^^37^ Expression of CD146 largely overlapped with CD105 within the tumor (Figure 5H, arrowheads) but was expressed at considerably lower levels on blood vessels of the surrounding normal brain parenchyma (Figures 5H–5J), pointing to the well described implication of this VEGFR2 co-receptor in tumor neo-angiogenesis.^38^ EphA2 has been described as an angiogenic signal in various solid tumors, expressed in both the cancer cells themselves and the newly generated endothelium.^39^ In support of these findings, we observed the presence of EphA2 on CD105^+^/CD146^+^ blood vessels within the tumor (Figure 5I, arrowheads) as well as diffuse staining in the surrounding GBM tissue. As expected, EphA2 was quasi-absent from normal brain tissue outside the tumor area (Figures 5I and 5J).

Next, we used the MICS data to study tumor cell invasion, another important hallmark of GBM. White matter tracts, in particular the CC, represent the most prominent infiltration routes in GBM.^40^^,^^41^ In agreement, MICS analyses identified the presence of a stream of mCherry^+^ cells integrated in the NF- and L1-CAM-positive fiber tract of the CC (Figures 5K and 5L). The absence of mCherry^+^ cells in the CC during the initial stages of tumor induction by LWE (Figure 2) indicates that SVZ-derived cancer cells migrate through this structure to invade adjacent tissues, as has been described in humans. Note also the presence of several CD11b^+^ myeloid cells in the CC, in proximity to cancer cells (Figure 5L, arrow). In addition to this massive stream in the CC, individual cells were observed distant from the lateral tumor margin in a pattern suggesting invasion of the neighboring striatum (Figure 5M). Interestingly, these mCherry^+^/EGFR^+^ cancer cells were not evenly distributed. Indeed, they were scarce in the region comprising mainly NeuN^+^ neurons, but accumulated along and inside the NF^+^ striatal axon bundles, in further support of a white matter-guided mode of tumor cell invasion.

Finally, we observed another typical mode of GBM tissue invasion via the brain vasculature. Indeed, we found that two main CD105^+^ arteries, localized in the septum, on the opposing side of the lateral ventricle, thus in considerable distance from the main tumor (Figures 5K–5N), were populated with mCherry^+^/EGFR^+^ cancer cells (Figure 5N, white arrows). It is notable in this context that several CD169^+^ bone marrow-derived macrophages (Figure 5N, blue arrows) were also present on the cancer cell-covered blood vessels, again indicating early interaction between invasive cancer cells and immune cells.

Altogether, the combined use of classical histological approaches, light sheet 3D microscopy, and ultrahigh content imaging by MICS provides deep insight into brain cancer development in this mouse model, exhibiting dense and morphologically complex tumors that show widespread invasion into healthy regions of the brain. These combined analyses strongly indicate that the model faithfully reproduces most cellular and molecular processes observed in human GBM. This work also provides a resource for mouse-compatible antibodies.

### Combining 3D light sheet microscopy with 2D MICS

Our 3D light sheet microscopy revealed that the induced mouse GBMs were highly complex and spread widely across the rostral-caudal and medio-lateral axes of the analyzed brains (Figure 3). In parallel, our molecular studies using MICS showed a high degree of molecular and cellular complexity, which could only be examined on individual tissue sections in standard planes, without considering the full three-dimensional extent and morphological complexity of the induced tumors. Our aim was to reconcile these two aspects by performing high-resolution MICS analyses of brains that had previously been analyzed by tissue clearing and LSFM. For these analyses, we switched from the water-based CUBIC clearing protocol to the organic solvent-based Miltenyi clearing kit. Although this change did not allow direct imaging of fluorescent proteins in LSFM, it provides better tissue stiffness, which allows higher-magnification imaging and shows better compatibility with the subsequent thin-sectioning needed for MICS analyses.

**Figure 6:**
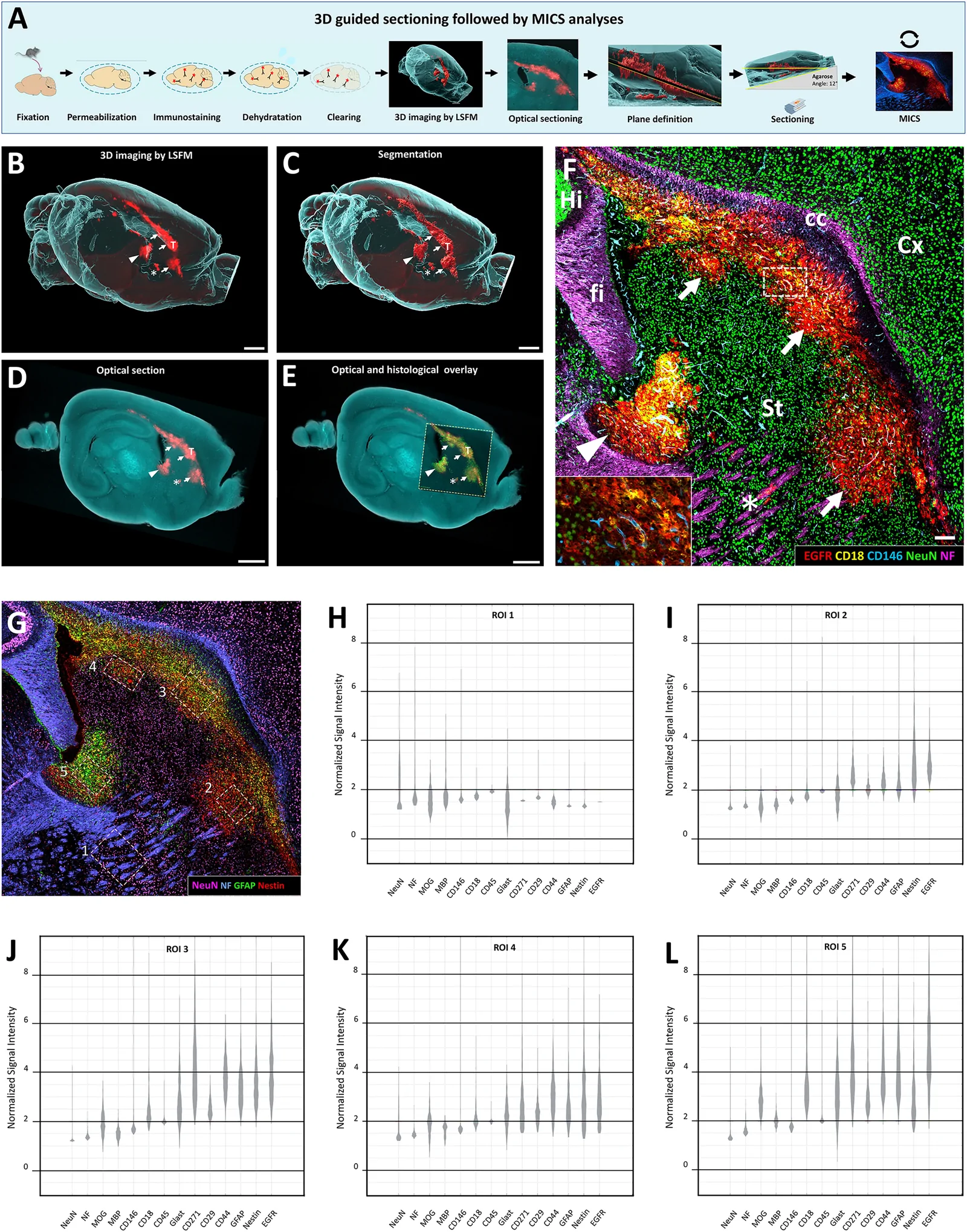
Sequential light sheet microscopy guided sectioning and MICS (A) Scheme of the protocol used for guided sectioning after imaging by LSFM followed by MICS analyses. (B) 3D raw image of a brain 2 weeks post-electroporation. The main tumor (T) extends into the neighboring parenchyma (arrows). A small group of cells detached in the striatum (asterisk) and a large non-connected tumor cell mass was observed along the ventral aspect of the ventricle (arrowhead). (C) Corresponding 3D view with segmented tumor-positive signal. For animation, see Video S5. (D) Optical section of the 3D view used to determine the optimal target plane. Tumor is shown in red and tissue autofluorescence in cyan. (E) Overlay of the selected optical section with a MICS image (green) of the corresponding tissue section. (F) High-resolution histological analyses using ultrahigh content imaging on a section generated by the 3D-2D protocol. Selected markers showing labeling of the cancer cells (EGFR), leukocytes (CD18), blood vessels (CD146), neurons (NeuN), and axonal fiber tracts (neurofilament, NF). (G–L) Quantification of marker expression in five different ROIs (shown in G) positioned either in the striatum (ROI1) or different regions of the tumor (ROI2-5). (H–L) Violin plot representation of expression levels for 14 tumoral and non-tumoral markers in the five ROIs. Number of segmented cells for each ROI: 471 cells (ROI1), 412 cells (ROI2), 398 cells (ROI3), 375 cells (ROI4), and 473 cells (ROI5). Scale bars, 1000 μm (B and C), 150 μm and 80 μm in inset (D). cc: corpus callosum, Cx: cortex, fi: fimbria, Hi: hippocampus, and St: striatum.

We induced experimental GBM by LWE, but expressed cytoplasmic GFP instead of nuclear mCherry, to allow visualization of cell morphology and detection with anti-GFP nanobodies. After perfusion at 2 wpe, brains were permeabilized, stained with an anti-GFP nanobody conjugate, and cleared using the MACS® Deep Clearing Kit (Figure 6A). 3D images were acquired, and surfaces were generated with IMARIS software (for detail, see Bigott et al.^42^). This approach allowed the identification and imaging of GFP-positive tumors at high quality (Figures 6B and 6C, 3D-animation in Video S5; brain surface in cyan, GBM in red). The main tumor (T) covered the wall of the lateral ventricle, underlying the CC and extending connected groups of cells into the underlying striatum (Figures 6B and 6C; arrows). In addition, a large group of GFP-expressing cells was identified in the ventral aspect of the lateral ventricle, separated from the main tumor (Figures 6B and 6C; arrowhead). Finally, a smaller isolated cluster of GFP-high cells was localized in the striatum (Figures 6B and 6C, asterisk; see Video S5).

Video S5. 3D view of a segmented tumor at 2wpe that was analyzed in the 3D–2D approach

Based on these 3D data, we used optical sectioning to define an optimal plane that covers the maximum extent of the tumor, including the isolated cell clusters, in a single histological section (Figure 6D). For the presented brain, we defined a 12° deviation from the mid-sagittal plane as optimal and established a protocol allowing precise cryostat sections at this angle (Figure 6A;^42^). After re-hydration, cryoprotection, angle correction, and snap freezing, a 10 μm-thick section at the desired level was selected. Overlay between the projected image based on LSFM (Figure 6D) and the physical section subjected to MICS was perfect (Figure 6E).

After MICS, 13 of the 38 markers used in the 2D-only MICS approach (Table 1) provided high-quality staining (Table 2, individual images in Figure S4). In addition, the low-affinity neurotrophin receptor CD271(p75),^43^ which did not provide high-quality staining in the MICS-only analyses, showed excellent and specific staining in the 3D2D approach and was further analyzed.

**Table 2:** Antibodies panel for MICS on 2D3D

Antigen	Clone	Dilution	Order no.	Fluorochrome	Company
CD18	REA1226	25	130-124-120	APC	Miltenyi Biotec
CD29	REA1074	50	130-119-165	PE	Miltenyi Biotec
CD44	REA664	100	130-119-121	APC	Miltenyi Biotec
CD45	REA737	50	130-110-796	FITC	Miltenyi Biotec
CD54 (ICAM-1)	REAL289	25	130-115-986	APC	Miltenyi Biotec
CD146 (LSEC)	REA1064	50	130-118-406	FITC	Miltenyi Biotec
CD271 (LNGFR)	REA648	25	130-118-793	PE	Miltenyi Biotec
EGF Receptor	REA939	200	130-115-506	APC	Miltenyi Biotec
GFAP	REA335	50	130-118-351	PE	Miltenyi Biotec
GLAST (ACSA-1)	ACSA-1	100	130-118-344	PE	Miltenyi Biotec
Mog	REAL797	25	130-128-468	PE	Miltenyi Biotec
Nestin	REA575	50	130-120-164	APC	Miltenyi Biotec
NeuN	REA1131	50	130-119-492	FITC	Miltenyi Biotec
Neurofilament	REA1127	50	130-119-496	PE	Miltenyi Biotec

Figure 6F shows a representative image with superposed staining for cancer cells (EGFR), immune cells (CD18), blood vessels (CD146), neurons (NeuN), and axonal projections (NF). In agreement with the 3D projection (Figures 6B and 6C), a main EGFR^+^ tumor mass was positioned underlying the CC (labeled by NF, magenta) with little to no invasion into the overlying cortex (NeuN) but extending deep into the striatum (arrows). In addition to the main tumor mass, a large cluster of eGFP^+^/EGFR^+^ cells was detected along the ventral aspect of the lateral ventricle (Figures 6B–6F; arrowheads). Finally, as expected from the 3D data, a smaller group of EGFR^+^ cells was found separated from the main tumor and integrated in the axon bundles of the striatum, suggesting tissue invasion from the main tumor by a white matter route (Figures 6B–6F, asterisk). Overall, correspondence between the tissue section and the tumor as detected by LSFM was excellent.

We quantified the expression of the 14 markers across distinct tumor regions and the underlying normal striatum. Following segmentation and *Z* score normalization using MACSiQ, we defined five ROIs (Figure 6G) and analyzed marker intensity at the cell level. Cells in normal striatal tissue (ROI 1) showed strong expression of neuronal and glial markers—including NeuN, NF, MOG, MBP, and GLAST—consistent with healthy parenchyma (Figures 6G and 6H). In contrast, cells within different tumor regions (ROIs 2–5) displayed high levels of transgenic overexpressed EGFR, as expected, together with robust expression of canonical tumor markers such as CD44, CD29, CD271, GFAP, and Nestin (Figures 6G and 6I–6L). Notably, these predefined tumor regions exhibited pronounced spatial heterogeneity. For example, GFAP and Nestin showed opposing expression patterns, with GFAP-low/Nestin-high profiles in ROI 2 (Figure 6I) and GFAP-high/Nestin-low profiles in ROI 5 (Figure 6L). CD271 showed strong expression in ROIs 3 and 5 (Figures 6J–6L), but comparably low levels in ROIs 2 and 4 (Figures 6I and 6K). Altogether, this highlights region-specific molecular states of cells in the overall tumor mass.

**Figure 7:**
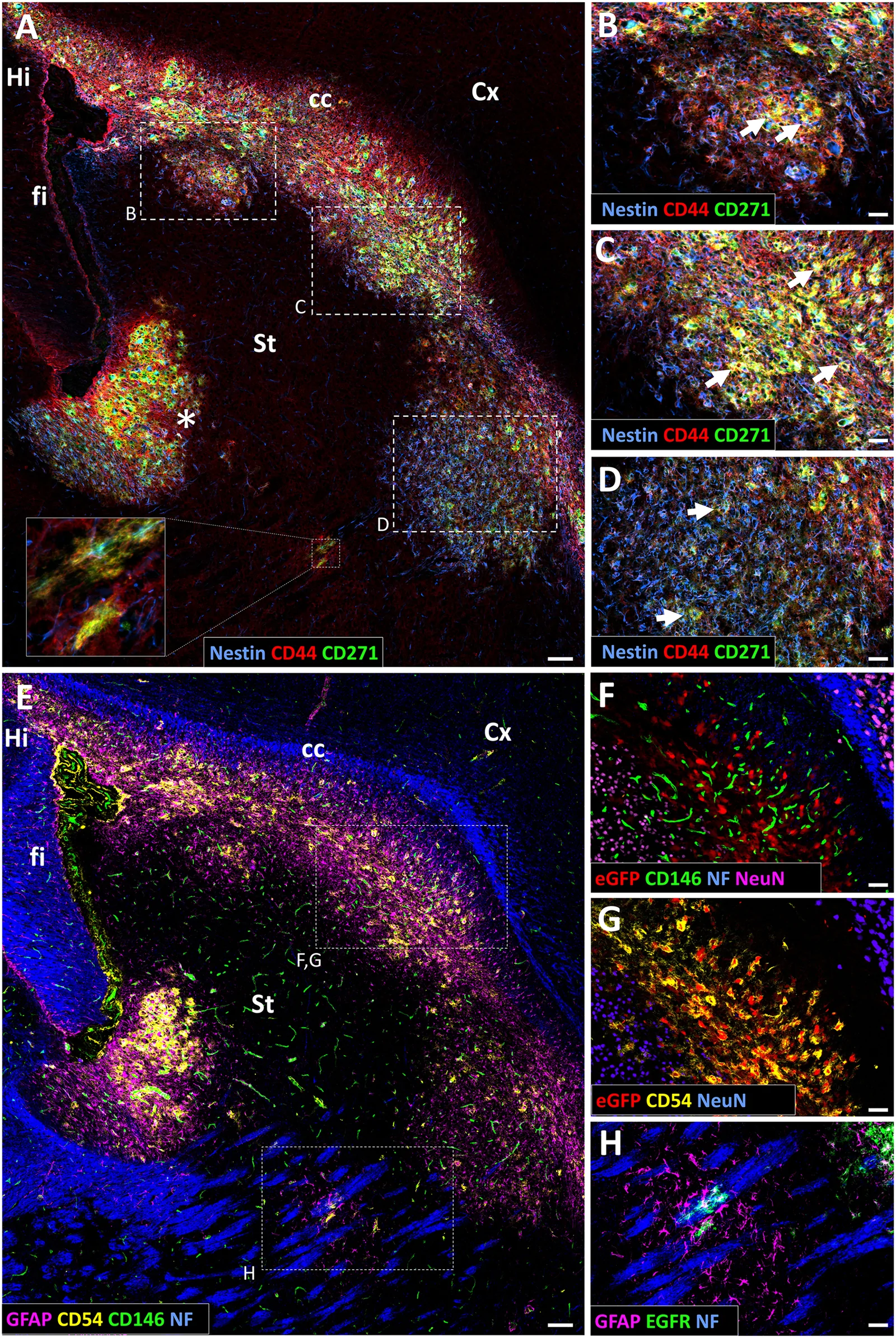
Ultrahigh-content imaging of 3D pre-analyzed tissue The same section as shown in Figure 6F was analyzed in different cellular and molecular contexts using relevant marker combinations. (A–D) High-resolution image showing differential spatial labeling for the tumor markers Nestin, CD44 and CD271. CD271, a marker for cancer stem cells and invading cancer cells, is confined to subpopulations in the main tumor (high magnification in B–D, arrows) and is expressed by invading cells in the striatum (insert in Figure 6A). (E–H) High-resolution images show staining for cancer cells (GFAP, CD54 (ICAM1), EGFR), vasculature (CD146), fiber tracts (NF), and neurons (NeuN). Boxed areas are presented at high magnification in F–H. In F and G, cancer cells are labeled with eGFP. In H, cancer cells are identified by high EGFR levels. Note GFAP-positive cells surrounding EGFR-positive cancer cells, indicating the presence of reactive astrocytes. Scale bars, 100 μm (A and E), 50 μm (B–D and F–H)

Next, we observed the cellular and tissue expression of classical markers for cancer cells in 3D pre-defined regions. Nestin was widely and homogeneously expressed in all regions of the induced tumor (Figures 7A–7D). Overlap of Nestin with CD44 expression was strong in regions of the main tumor that were localized in proximity to the CC (Figure 7C). Extensions of the main tumor into the underlying striatum were also strongly Nestin-positive but showed overall lower CD44 expression levels (Figures 7B–7D).

As mentioned, another tumor marker that provided high-quality staining was CD271(p75), a marker for tumor-initiating cancer stem cells as well as for highly infiltrative cells that emigrate from the main tumor and thereby evade surgical resection.^44^ Indeed, high expression levels in humans associate with high-grade glioma and poorer overall survival.^30^^,^^44^ MICS analyses showed expression of CD271 in clusters of Nestin^+^/CD44^+^ cells in all regions of the induced tumor (Figures 7A–7D, arrows). However, the number of CD271 expressing cells was heterogeneous in different tumor regions, ranging from merely individualized cells in areas extending into the striatum (Figures 7A, 7B, 7D, and 6I–6K), to a predominance of CD271^+^ tumor cells in proximity to the CC (Figures 6J and 7C) and in the group of tumor cells positioned at the ventral aspect of the lateral ventricle (Figure 7A, asterisk, Figure 6L). Interestingly, CD271 was also expressed in the isolated cluster of cells integrated in the striatal axon bundles (see insert in Figure 7A), further arguing that this group is a result of tissue invasion.

Other examples of markers that showed high-quality staining in the 3D2D approach were CD146, CD54, and GFAP (Figures 7E–7H). Indeed, comparable to the 2D-only MICS analyses (Figure 4G), CD146 was a reliable marker of blood vessels in the tumor and at lower levels in the surrounding normal brain (Figures 7E and 7F). CD54, a potent cancer cell-secreted chemoattractant for immune cells^45^ colocalized with GFP cancer cells but was absent from the normal brain tissue (Figures 7E–7G). As expected, GFAP was strongly expressed in the different tumor regions, largely overlapping with the presence of Nestin and CD44 (Figure 7E, compare Figure 7A). Interestingly, strong GFAP^+^ cells with astroglial morphology were also detected surrounding the above-described separated cluster of EGFR^+^ cancer cells in the striatum (Figures 7E–7H), indicating a rapid inflammatory response of the surrounding brain parenchyma. Altogether, these analyses show that tissue clearing and 3D imaging are compatible with the subsequent high-content spatial imaging approach provided by MICS, permitting molecular analyses of pre-localized brain regions.

## Discussion

Deep characterization of preclinical models—enabled by a comprehensive toolbox—is essential for their effective use and for translating experimental findings into clinical applications. In this work, we integrate classical histology with cutting-edge 3D imaging and spatial proteomics to characterize a clinically relevant electroporation-based glioblastoma (GBM) mouse model.

By combining CRISPR/Cas9-mediated mutagenesis of *TP53* and *PTEN* with transposase-driven genomic integration of the *EGFRvIII* oncogene, we establish a simple and reliable model that meets all histopathological criteria for high-grade glioma. Unlike traditional approaches, this model can be applied across virtually any genetic background in mice, eliminating the need for laborious and time-consuming generation of complex allelic combinations.

The model’s rapid onset and high temporal precision enable the study of events as early as two days post-transformation of normal NSCs. Using tissue clearing and 3D LSFM, we demonstrate that tumor growth is highly reproducible and quantifiable across cohorts and extended time frames, detectable as early as one week after transformation. Additionally, multiplex tissue imaging allows us to dissect the molecular and cellular composition of induced tumors. We provide a curated list of mouse-compatible antibodies for high-resolution analysis of GBM and its micro- and macroenvironments. Finally, we show that tissue clearing and LSFM are compatible with subsequent multiplexed spatial proteome analysis via MICS technology, enabling serial 3D-to-2D analyses.

Over the past decade, numerous mouse models have been developed to study high-grade gliomas, offering insights into GBM biology and its interactions with healthy tissue.^8^^,^^9^^,^^13^^,^^14^^,^^46^ Many of these models rely on gain-of-function *EGFR* mutations, a potent driver of neural stem cell proliferation, migration, and differentiation, and a key GBM oncogene^3^ as well as a promising target for CAR-T cell therapies.^47^ Unlike other *EGFR*-based models—where tumor induction and progression span weeks or months^48^ —our model achieves rapid and homogeneous tumor induction, likely due to high *EGFRvIII* expression driven by the actin-based *CBh* promoter. Nevertheless, our histopathological analyses confirm that the resulting tumors exhibit all hallmark features of high-grade glioma. Moreover, the speed and reproducibility of tumor induction grant unprecedented access to the earliest stages of NSC transformation. Here, we show that exit of transformed cells from progenitor zones occurs very early, independent of tumor mass growth. The model’s homogeneous and quantifiable growth—validated by LSFM—and its compatibility with diverse genetic contexts make it a powerful tool for studying the molecular regulation of brain cancer development and for preclinical testing of therapeutic approaches.

A major advantage of somatic GBM mouse models, such as the one presented here, is that tumor development occurs in a fully immunocompetent environment. In humans, immune cells constitute up to 40% of the tumor mass, comprising primarily brain-resident microglia and bone marrow-derived myeloid cells.^49^ The prevailing hypothesis suggests that myeloid cells are initially recruited to eliminate cancer cells but are reprogrammed to support tumor growth. However, most supporting evidence comes from late-stage human tumors or fully established mouse models, overlooking the critical early phases of immune involvement.^50^^,^^51^^,^^52^ Combining somatic models that enable controlled progression from early gliomagenesis to lethal tumors with deep molecular phenotyping via spatial proteomics offers a robust approach to advance this therapeutically relevant field. In the model, we observe that as early as two weeks post-induction, mouse tumors contain abundant CD11b^+^/CD45^+^ cells.^53^ We also identify a significant immune subpopulation expressing CD169, representing blood-derived myeloid cells that contribute to anti-tumor immunity.^14^ Additionally, a subset of CD11b^+^/CD45^+^ myeloid cells expressed CD11c, a marker of primed microglia and infiltrating monocyte-derived dendritic cells,^54^ either alone or in combination with CD169. While this marker set is not exhaustive, it provides a foundation for deep spatial proteomic analyses of tumor-immune interactions in somatic mouse models.

A central aim of this work was to provide new tools and concepts for the use of high-content multiplex imaging to molecularly characterize induced mouse gliomas. While multiplex spatial proteomics via MICS has been successfully used in human tissues,^15^^,^^55^ its application in mouse GBM research remains limited.^56^ To our knowledge, multiplex spatial imaging has not previously been applied to somatic mouse models like the one described here.

One major obstacle for the use of the multiplex approach is the scarcity of validated antibodies for mouse tissues. Indeed, most available antibodies—targeting cancer cells or components of the micro- and macroenvironments—were developed and optimized for human samples. Consistent with this, we screened an initial panel of 77 human-validated antibodies and found that 38 produced high-quality staining in mouse GBM sections. While this provides a useful starting point, it is neither comprehensive nor unbiased, highlighting the need for systematic marker selection and validation across additional biological contexts. Nevertheless, the antibodies we identified cover nearly all relevant tumoral and non-tumoral cell subpopulations. Canonical brain tumor markers—such as GFAP,^57^ nestin,^29^ CD44,^58^ and GLAST^33^—were strongly expressed in cancer cells of tumor-bearing regions, with patterns and cellular localizations that further validated the clinical relevance of our model. We also detected CD271, a marker functionally linked to chemoresistant cancer stem cells and infiltrative cancer cells that evade surgical resection in human GBM.^44^ In our mouse model, CD271 was strongly expressed not only in subsets of cells within the tumor core but also in cells migrating along white matter tracts, mirroring the invasive behavior observed in patients.

The integration of tissue clearing, 3D microscopy, and targeted 2D multiplex imaging in one workflow represents a significant technical advance with broad applications. Indeed, 3D microscopy enables the detection of a limited marker set within its true spatial context.^59^ 2D multiplexing provides deep molecular and cellular insights—but typically from randomly selected sections in standard anatomical planes (coronal, sagittal, or horizontal). By leveraging high-resolution 3D images, we defined optimal target planes to prepare tissue sections precisely aligned with regions of interest—a workflow we term light-sheet-guided sectioning.^42^ This method ensures that sections capture multiple pre-identified tumor regions, enabling direct comparisons within the full 3D context. Moreover, since MICS is time-consuming and costly, our 3D-to-2D approach optimizes efficiency by pinpointing the ideal tissue section that provides maximum information in a given experimental context. Critically, it also allows the identification of invading cells distant from the tumor core, which would likely be missed in conventional histological sections. Thus, light-sheet-guided sampling maximizes spatial information recovery and enhances analytical precision.

In the subsequent MICS analyses of predefined tissue sections 13 of the initially identified 38 markers (plus CD271) yielded high-quality staining for both cancer cells and the tumor micro- and macro-environment. Although this panel is limited, all major cell populations present in brain tumors could be identified, confirming the feasibility and usefulness of the approach. A systematic expansion—testing a broader spectrum of markers, as well as variables such as fixation methods and antibody concentrations—will likely yield a far more comprehensive list of antibodies suited for sequential 3D-to-2D analyses.

Previous studies showed that CUBIC-cleared, LSFM-analyzed human tissues retain antigenicity for classical immunohistochemistry.^60^ However, because classical staining is low-throughput, only a limited number of markers could be assessed, restricting the analysis of complex mechanisms and interactions, as can be performed with a high-throughput analytic system such as MICS. Alternatively, approaches such as MS-DISCO^61^ enable large-scale and systematic proteome analysis of tissues after 3D imaging, by using LSFM to identify regions of interest (ROIs), which are then isolated via robotic extraction and analyzed by mass spectrometry. While such an approach provides unbiased proteome data for predefined regions, it lacks cellular or subcellular resolution—a limitation overcome by our LSFM/MICS workflow.

Thus, combined use of 3D light-sheet imaging and 2D multiplex proteomics provides a powerful framework for spatially resolved molecular analysis of somatic high-grade mouse gliomas. Despite limited availability of rodent-validated antibodies, we identify a functional marker set and show that light-sheet-guided sampling enables precise, information-rich sectioning. This integrated workflow expands the toolkit for mouse GBM research and establishes a practical path toward systematic spatial proteomic characterization of tumor ecosystems. It is evident that this approach is not limited to cancer biology, but that a wide spectrum of applications, ranging from developmental biology research to various disease contexts, can be envisioned.

### Limitations of the study

• In this manuscript, we characterize a specific somatic mouse GBM model based on *p53/PTEN* mutagenesis and *EGFRvIII* overexpression, which is distinguished by its very rapid growth. While this focused analysis provides a clear view of the model’s properties, it would undoubtedly be highly informative to perform a comparative evaluation of different electroporation-based GBM models in parallel, as this would help determine how generalizable the findings are across varying genetic contexts and tumor behaviors.
• A limitation of this work concerns the size and content of the marker panel used for the MICS analyses. Among the initially tested 77 antibodies in the 2D MICS workflow, all sourced from Miltenyi Biotec, 38 yielded staining quality suitable for in-depth analysis in mouse tissue. While this represents a substantial set, it is not comprehensive or unbiased, and systematic marker selection and validation will be required across different biological contexts. Moreover, in our combined 3D–2D workflow, 14 markers proved robust staining under our experimental conditions. This panel was sufficient to demonstrate proof-of-principle and to extract biologically meaningful information in the context of tumor analysis. However, it is clear that expanding the antibody repertoire and optimizing alternative fixation and staining protocols will be essential to enable systematic and versatile application of the technology to a broader range of biological questions.
• In this study, we used the CUBIC clearing protocol for direct imaging of mCherry and shifted to the commercial Miltenyi kit for the 3D2D approach. In principle, the MICS staining protocol should be compatible with CUBIC clearing, but we did not systematically test this possibility.

## Resource availability

### Lead contact

Requests for further information and resources should be directed to and will be fulfilled by the lead contact, Marie-Catherine Tiveron (marie-catherine.tiveron@univ-amu.fr).

### Materials availability

Plasmids generated in this study will be made available on request, but we may require a completed material transfer agreement if there is potential for commercial application.

### Data and code availability

• Data: Data reported in this paper will be shared by the lead contact upon request.
• Code: This paper does not report original code.
• All other items: Any additional information required to reanalyze the data reported in this paper is available from the lead contact upon request.

## Acknowledgments

We thank JH Lee (KAIST, Daejeon, South Korea) and Karine Loulier (INM, Montpellier, France) for CRISPR and pBASE plasmids, respectively. Caroline Blanc is acknowledged for help with experimental work. The Cremer lab has been supported by Association pour la Recherche contre le Cancer (ARC) and the Cancéropole PACA. Yliana Huriaux Fontana was supported by a PhD fellowship from the 10.13039/501100002915Fondation pour la Recherche Médicale (FRM). We also thank the imaging facility at IBDM, a member of the National Infrastructure France-BioImaging (https://ror.org/01y7vt929) supported by the 10.13039/501100001665French National Research Agency (ANR-24-INSB-0005 FBI BIOGEN) as well as the animal facilities. The Tchoghandjian lab was supported by ANR (MEGA01, ANR-21-CE18-0047-01), the 10.13039/501100014182Cancéropole PACA (PETRA structuring action) and the patient association pour la recherche sur les tumeurs cérébrales (ARTC Sud).

## Author contributions

Conceptualization: H.C., M.C.T., N.C., A.B., K.B., and A.T.; methodology: M.C.T., K.B., N.C., H.C., F.E.Y., S.R., M.J., and D.F.B.; experimental work: M.C.T., N.C., K.B., V.S., Y.H.F., M.C., L.V., and F.E.Y.; analyses: M.C.T., N.C., H.C., F.E.Y., K.B., V.S., D.F.B., and A.T.

## Declaration of interests

K.B., V.S., S.R., A.J., and A.B. are employees of Miltenyi Biotec B.V. & Co.

## Declaration of generative AI and AI-assisted technologies in the writing process

During the preparation of this work, the authors used AI tools exclusively for text editing. The authors reviewed and edited the content as needed and take full responsibility for the content of the publication.

## STAR★Methods

### Key resources table

**Table:** 

REAGENT or RESOURCE	SOURCE	IDENTIFIER
**Antibodies**		

Anti-Olig2 Clone EP112 (Rabbit igG)	Zytomics	Cat#: BSB2563
Anti-Ki-67 antigen clone MIB-1 (Mouse IgG1, kappa)	DAKO	Cat#: GA626; RRID: AB_2687921
anti-GFP Alexa Fluor 647 nanobody	Proteintech/ChromoTek	Cat#: gb2AF647-50; RRID: AB_2827575

**Chemicals, peptides, and recombinant proteins**		

Heparin sodium salt	Sigma-Aldrich	CAS: 9041–08-1
EdU	Sigma-Aldrich	CAS: 61135–33-9
Azide	Sigma-Aldrich	CAS: 26628–22-8
Paraformaldehyde 32% solution, EM grade	Electron Microscopy Sciences	CAS: 30525-89-4
Tissue-Tek OCT Compound	Electron microscopy Sciences	Cat #: 62550–01
Mowiol 4–88; Poly(vinyl alcohol)	Sigma-Aldrich	CAS: 9002-89-5
Hoecsht H33258 Solution	InVitrogen	CAS: 23491-45-4
Urea	Euromedex	CAS: **57-13-6**
Triton X-100	Euromedex	CAS: 9036-19-5
*N*,*N*,*N*′,*N*′-tetrakis-(2-hydroxypropyl)ethylenediamine	Sigma-Aldrich	CAS: **102–60–3**
TOPRO-3 iodide nuclear dye solution	Life Technologies	CAS:157199-63-8
PBS	Eurobio	Cat#: EM-15714
Low melting agarose	Sigma-Aldrich	CAS: 39346–81-1
Sucrose	Euromedex	CAS: 57–50-1
Absolute Ethanol	VWR	CAS: 64-17-5
Tween® 20	Euromedex	CAS: 9005-64-5

**Critical commercial assays**		

Click-iT EdU imaging kit	Life Technologies	Cat#: C10340
MACS® Deep Clearing Kit	Miltenyi Biotec	Cat#:130-136-470

**Experimental models: Organisms/strains**		

Wild type Rj:Swiss CD1 mice	Janvier labs	N/A

**Recombinant DNA**		

phU6-sgTP53-sgPTEN-Cbh-Cas9-Cre plasmid	DR. Jeong Ho Lee, KAIST, South Korea	Lee et al.^3^; https://doi.org/10.1038/s41586-018-0389-3.
pCAAGS hyPBase plasmid	Dr. Karine Loulier	N/A
pPB-CMV-TO-EGFRvIII-IRES-nlsCherry	DR. Michael Elowitz	RRID: Addgene_116039

**Software and algorithms**		

ImSpector software	Miltenyi Biotec	N/A
IMARIS (version 10.2.0)	Oxford Instruments, Zurich, Switzerland	https://imaris.oxinst.com
GraphPad Prism (version 9.0)	Prism GraphPad Software, USA	https://www.graphpad.com
NDP.view2 Software	Hamamatsu Photonics K.K, Japan	https://www.hamamatsu.com
R version 4.4.2	The R Foundation	https://www.r-project.org/; RRID: SCR_001905
R Package RcmdrPlugin.coin	The R Foundation	https://CRAN.R-project.org/package=RcmdrPlugin.coin
FIJI software	ImageJ2	https://imagej.net/ij/; RRID: SCR_003070
MACSiQ View Sofware	Miltenyi Biotec	N/A
ZEN software	Zeiss, Germany	https://www.zeiss.com

### Experimental model and study participant details

Experiments were exclusively performed in mice. All experiments were approved by the ethical committee of the French Ministry of Higher Education, Research and Innovation (Authorization number: 34083-2021112215573540v5), based on European Community rules and regulations.

Rj:Swiss CD1 mice (Charles River, France) were group-housed in regular cages under standard conditions (temperature: 22°C + 2; hygrometry: 50–70%) on a 12 h light-dark cycle. Mice were free of pathogens except for helicobacter and norovirus. The day of birth was defined as postnatal day 0 (P0) and histological analyzes were performed at weekly intervals up to 6 weeks of age. For survival assays mice were monitored daily and euthanized when they began to show signs of disease (hunching, limited activity). Both male and female were used in all experiments.

### Method details

#### Plasmid construction

The two vectors we used to induce high grade glioma were constructed as follows. The vector that allows the expression of the CRISPR Cas9 system together with the PiggyBac transposase, was generated from phU6-sgTP53-sgPTEN-Cbh-Cas9-Cre plasmid (kind gift from Dr. Jeong Ho Lee, KAIST, South Korea),^3^ in which the sequence of Cre recombinase (Cre) was replaced by that of hyperactive PiggyBac transposase (hyPBase)^62^ from pCAAGS hyPBase (phyPBase, kind gift from Dr. Karine Loulier). The PiggyBac expression vector, that carries *EGFRvIII* and nuclear *mCherry*, was obtained from pPB-CMV-TO-EGFRvIII-IRES-nlsCherry (gift from Michael Elowitz, Addgene plasmid # 116039) by replacing the CMV promoter with the Cbh promoter^63^ (PB-EGFRvIII-mCherry) This construct was further modified to replace *nlsCherry* gene by *eGFP* sequence to obtain pPB-Cbh-EGFRvIII-IRES-eGFP (PB-EGFRvIII-GFP). For control experiments, the *EGFRvIII* sequence was deleted from PB-EGFRvIII-mCherry and PB-EGFRvIII-GFP. These new recombined plasmids were combined individually with phU6-sgTP53-sgPTEN-Cbh-Cas9-hyPBase for use in electroporation experiments to prevent tumor induction.

#### Postnatal electroporation

*In vivo* electroporation was performed as previously described.^4^ Briefly, P0-P1 pups were anesthetized by hypothermia and injection of 1.5 μL of a mixed solution of each plasmid at 1 μg/μL were injected in the lateral ventricle. Animals were subjected to five 95V electrical pulses (50 ms, separated by 950 ms intervals) using the CUY21 edit device (Nepagene, Chiba, Japan) and 7 mm tweezer electrodes (CUY650P7, Nepagene) coated with conductive gel (Signagel, Parker Laboratories, USA). To target effectively the dorsal or lateral V-SVZ, the positive electrode was positioned respectively, dorsally above the eyes or slightly ahead of the eyes, and the negative electrode was placed in the opposite position. After electroporation, animals were reanimated in a 37°C incubator before returning to the mother.

#### Brain tissue preparation

Mice were deeply anesthetized with a lethal dose of xylazine and ketamine (200 and 20 mg/kg respectively). Intracardiac perfusion was performed with cold PBS; heparin (5u/mL; H3393, Sigma-Aldrich), to wash out efficiently blood cells from the vasculature, followed by fixation with cold 4% paraformaldehyde in PBS (32% stock solution, Electron Microscopy Sciences). Brains were then dissected out, post fixed overnight then stored in PBS. To label rapid dividing cells, EdU solution (50 mg/kg; B5002, Sigma Aldrich) was injected intraperitoneally 1 h before perfusion. For MICS analyses, brains were post fixed for only 2 h, washed quickly in PBS, then cryoprotected in a 30% sucrose-PBS solution, embedded in OCT (Tissue-Tek) and finally frozen in isopentane cooled in dry ice. Samples were cryosectioned with a CM3050S cryostat (Leica) and 10 microns coronal sections were mounted on Superfrost glass slides and stored at −80°C until processed. For the combination of light sheet microscopy and MICS, brains were post fixed for only 2 h, washed quickly in PBS and stored in PBS containing sodium 0.09% azide (S2002, Sigma Aldrich).

#### Immunohistochemistry and histology analyses

For immunofluorescence analysis, fixed brains were cryoprotected in 30% sucrose, then sectioned with microtome (MICROM HM450). EdU incorporation was detected on 50 μm floating sections using Click-iT EdU imaging kit (C10340, Life Technology). Sections were finally incubated in Hoechst solution (1/2000; H3570, InVitrogen) and mounted on Superfrost glass slide in Mowiol® 4–88 (475904, Sigma-Aldrich). Optical images were acquired with an Axioplan2 ApoTome microscope (Zeiss, Germany) using ZEN software (Zeiss), and processing was performed using Fiji software. For histology, serial 5-μm formalin-fixed paraffin-embedded (FFPE) sections of mouse brains were stained with hematoxylin and eosin (H&E) and examined under the microscope for the presence of tumor cells. Immunohistochemistry was performed on adjacent paraffin sections. After steam-heat-induced antigen retrieval, sections of FFPE samples were tested for the presence of Olig2 (Clone EP112, Zytomics, dilution 1:100) and Ki-67 (Clone Mib-1, DAKO, dilution 1:200). A Benchmark Ventana autostainer (Ventana Medical Systems) was used for detection, and slides were simultaneously immunostained in order to avoid inter-manipulation variability. Slides were then scanned (Nanozoomer 2.0-HT, Hamamatsu Photonics SARL France, Massy, France) and images processed in NDP.view2 software (Hamamatsu).

#### Brain clearing and 3D imaging for tumor volume assessment

##### Clearing

Brains were cleared using the advanced Clear, Unobstructed Brain Imaging Cocktails (CUBIC) protocol.^25^ Fixed hemi brains recovered at 1, 2, 3 and 4 wpe were incubated in 7 mL of CUBIC reagent 1 [25% urea (Euromedex), 25% *N*,*N*,*N*′,*N*′-tetrakis-(2-hydroxypropyl)ethylenediamine (Sigma-Aldrich), and 15% Triton X-100 (Euromedex)] at 37°C under gentle shaking until complete clearing. CUBIC reagent 1 solution was changed after the first day of incubation and then every 2 days. When brains were transparent, they were washed for 1 h in PBS and then incubated in TOPRO-3 iodide nuclear dye solution (1:2000; T3605, Life technologies) overnight at 37°C. Brains were further washed in PBS 2–3 times for 10 min and immersed back in CUBIC reagent 1 for one more day at 37°C.

##### Light sheet imaging

Cleared brains were imaged in CUBIC1 solution in horizontal orientation with an Ultra Microscope Blaze (Miltenyi Biotec, Germany) controlled by Imspector Pro 7.3.2 and equipped with a sCMOS 5.5Mpx Camera, LaVision Bio Tec PLAN 1.1*x*/0.1 NA/≤16 mm WD objective and LED lasers at 488, 561 and 639 nm. Emission filters used were 525/50, 595/40, 680/30. Magnification lens 0.6× was used for larger brains at 4 wpe and 1*x* for all other samples, so they could be imaged in a single field of view. Samples were illuminated by the laser light sheet of 11.4 μm thickness, from one or both sides depending on the size of the samples. Images (TIFF format) were acquired with a z-step size of 6 μm, without dynamic focus. Stacks of images were used to perform 3D reconstruction.

##### Image analyses

3D projections were performed using Imaris software (version 10.2.0, Oxford Instruments, Zurich, Switzerland). The volume of tumors (V_Tumor_) was defined by creating a 3D mask based on mCherry labeling using the automatic surface tool of the Imaris software. Thresholding was performed using the background subtraction method for tumorsat 1 wpe and absolute intensity for the other stages. Threshold was tweaked manually and separately for each sample, to best fit with differences in background. Because the CUBIC protocol causes tissue swelling, tumor volume was normalized over the volume of the dentate gyrus (DG), a well-defined anatomical structure. The contours of the DG were drawn manually with the Surface tool using TOPRO3 signaling to generate a 3D mask and define the DG volume (V_DG_). Normalized tumor volume was obtained by dividing V_Tumor_ by V_DG_.

#### 2D MICS

##### Tissue preparation for MICS

Perfused brains were treated as described. Samples were cryo-sectioned with a CM3050S cryostat (Leica), 10 microns coronal sections were mounted on Superfrost glass slides according to the MACSwell™ Imaging Frame template and stored at −80°C until processing with MACSima™ imaging cyclic staining (MICS) technology.^15^^,^^55^

##### Antibodies and reagents for MICS

Primary fluorochrome-labeled antibodies conjugated to FITC, PE APC were used for MICS. Antibody clone used, their dilution and the conjugated fluorochrome are indicated in Table 1.

##### Cyclic staining

MACSima™ imaging cyclic staining was performed for deep cell phenotyping. Samples were mounted on the selected MACSwell™ Imaging Frame and rehydrated with MACSima™ Running Buffer for 2 min. DAPI pre-staining (1 μg/mL) was performed in MACSima™ Running Buffer for 10 min and washed three times with MACSima™ Running Buffer. Subsequently, MICS technology was applied with iterative staining, imaging and erasure cycles, enabling ultrahigh-content imaging on a single sample.^15^^,^^55^

#### 3D-2D workflow

A detailed protocol for the 3D-2D workflow was published in STAR Protocols.^42^

##### In short: 3D spatial analysis (LSFM)

Two weeks post electroporation with a phU6-sgTP53-sgPTEN-Cbh-Cas9-Cre-hyPBase/PB-EGFRvIII-GFP vector combination, brain hemispheres were stained and cleared according to MACS® Deep Clearing Kit protocol (Miltenyi Biotec, 130–136-470). Briefly, after perfusion, samples were permeabilized for 24 h in Deep Permeabilization Solution at room temperature, followed by immunostaining for 7 days at 37 °C with an anti-GFP Alexa Fluor 647 nanobody conjugate (Proteintech/ChromoTek, gb2AF647-50, 1:1000) and washed at room temperature, both in Deep Antibody Staining Solution. Dehydration was performed at 28 °C in an ascending ethanol series in distilled water (30%, 50%, 70%, 90%, 100%, 100%) with added 2% Tween 20 (v/v). Tissues were rendered transparent in high refractive index Deep Clearing Solution before imaging with an UltraMicroscope Blaze™. 3D light sheet data was acquired with both 1.1*x*/0.1 NA/≤17 mm WD and 12*x*/0.53 NA/≤10.9 mm WD objectives for overview and detail view, respectively. Fast imaging of detailed image stack was enabled by the combination of “Fast Tiling Scan” and “Speed Mode” within ImSpector software (Miltenyi Biotec).

##### 3D to 2D transition

Surfaces for both autofluorescence and anti-GFP Alexa Fluor 647 signal (GBM) were created within IMARIS analysis software. With these surfaces, an angle between mid-sagittal brain hemisphere surface and an optimal target intersection of the GBM could be determined via two “Oblique Slicer” planes. An agarose block (1.5% low melting agarose) with this specific angle was generated. Rehydration was performed at 28°C in a descending ethanol series in distilled water (100%, 90%, 70%, 50%, 30%) with added 2% Tween 20 (v/v), followed by two 1xPBS incubations. Samples were cryoprotected at 2°C–8°C in 30% sucrose-PBS solution until fully sedimented. Excess sucrose-PBS solution was removed and tissue were placed on top of the previously generated angled agarose block to harmonize the cutting surface and the GBM target section. Agarose block and tissue were carefully covered with optimal cutting temperature (OCT) compound within a cryomold and snap-frozen in liquid nitrogen cooled isopentane. Sample were stored at −80 °C until further processing.

##### 2D sample preparation

Samples were mounted in a cryostat as parallel to the blade as possible to intersect the surface fully, which ultimately preserved harmonization of surface and target plane. Sectioning was conducted at −20 to −23 °C and 8–10 μm slices were collected on SuperFrost® slides with a MACSwell™ frame template. The target region was verified with anatomical landmarks on a stereo microscope as well as by detection of preserved 3D-immunofluorescence signature with a fluorescence microscope.

##### 2D spatial analysis (MICS)

MACSima™ imaging cyclic staining (MICS) was performed as described before (Kinkhabwala et al., 2022).3 D-immunofluorescence signature of GBM was acquired in the APC-channel during autofluorescence acquisition.

Quantification: For quantification of marker expression tissue sections were segmented using MACSiQ and subjected to *Z* score normalization. Five ROIs in different regions of the tumor and the underlying striatum were defined, and the signal intensity of cells for all 14 markers was visualized as violin plots.

### Quantification and statisticals analysis

#### Quantification

Advanced cell segmentation was performed on the MICS image datasets using MACS iQ View software (Version 1.3.2) segmentation pipeline. The software uses nuclei and cell membrane markers to perform image segmentation identifying individual cells. Segmentation principles are described in Kinkhabwala et al.^15^

For expression analyses presented in Figure 4B we normalized data by *Z* score and defined four cell populations based on the strong expression of cell type-specific markers (NeuN, CD11b, CD105, mCherry). Using this pre-classification, we analyzed the protein expression of all 38 markers across the subsets, with results presented as a heatmap. All analytical steps were performed using the MACSiQ™ View software.

Vascular density was calculated as the surface area occupied by labeled pixels per region of interest (ROI). The analysis was performed with Fiji software. Twelve ROIs of identical size (24,858 μm^2^) were positioned either inside or outside the tumor, avoiding stitched regions. A labeling threshold was defined for each vascular marker (CD105, CD146, or EphA2), and pixels above this threshold were selected for analysis.

#### Statistical analyses

To analyze survival data the Kaplan-Meier method was used and statistical comparison between groups were performed with the Mantel-Cox (log rank) test using GraphPad Prism version 9.0 (GraphPad Software, USA). For histological analyses, in Figures 2G–2J and 5J, measurements are presented as mean ± standard error to the mean (sem). Statistical comparisons were performed with the nonparametric Wilcoxon-Mann-Whitney test, using R software. *n* values are presented in the figures legends. All *p* values less than 0.05 were considered statistically significant.
