# Supplementary material for: Integrated 3D light sheet and 2D multiplex imaging for deep histological characterization of somatic mouse glioblastoma
Source: iScience. 2026 Sep 8;29(9):117476. doi: 10.1016/j.isci.2026.117476 (PMC13573706; doi:10.1016/j.isci.2026.117476)
Supplement: Supplementary file 1 [file mmc1.pdf]

iScience, Volume *29*

Supplemental information

Integrated 3D light sheet and 2D multiplex imaging for deep histological characterization of somatic mouse glioblastoma

Marie Catherine Tiveron, Nathalie Coré, Kevin Bigott, Yliana Huriaux Fontana, Maria Caccavale, Léna Vilvandre, Fabio El Yassouri, Victoria Schoppel, Silvia Rüberg, Melanie Jungblut, Dominique Figarella-Branger, Aurélie Tchoghandjian, Andreas Bosio, and Harold Cremer

Figure S1

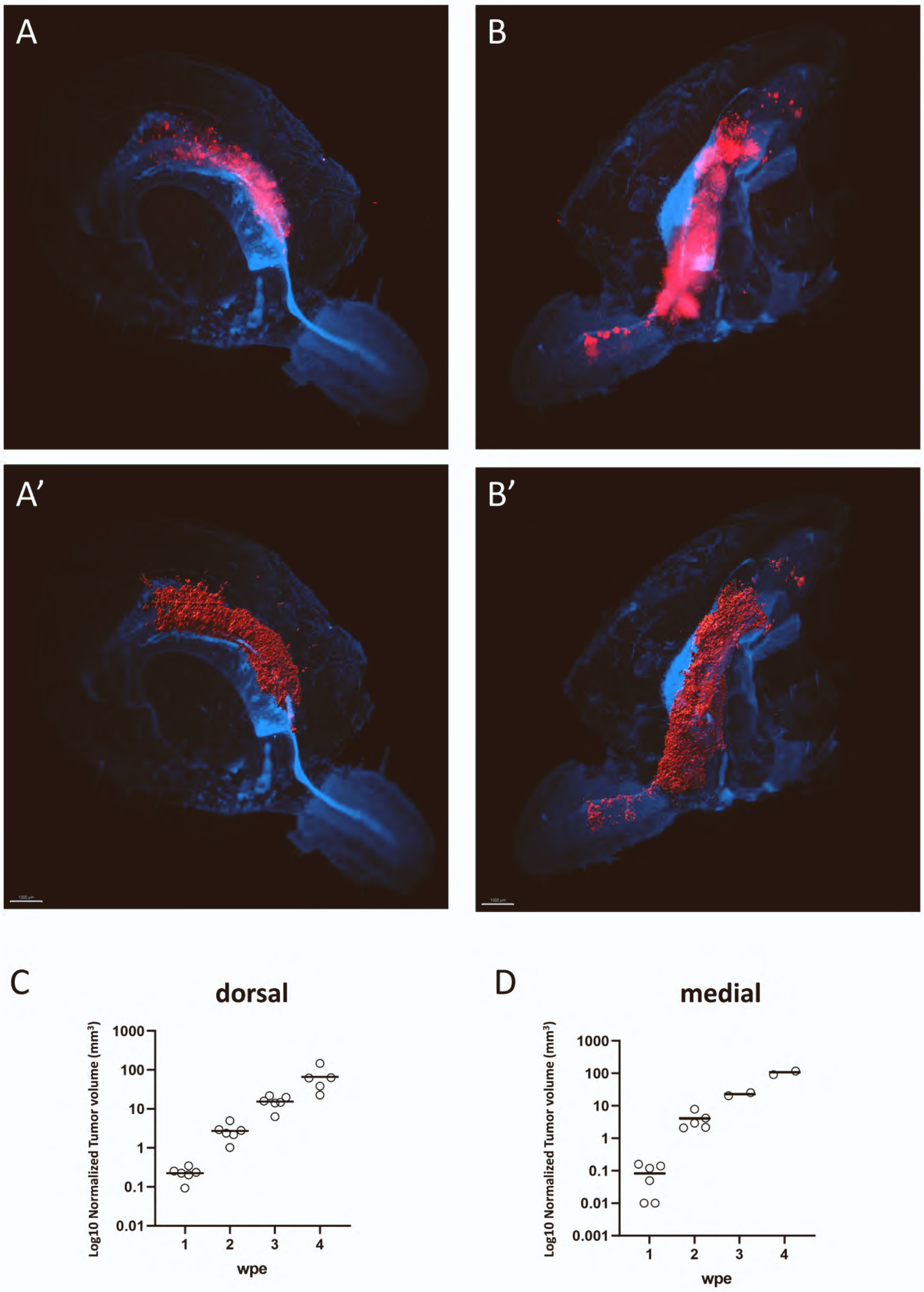

Supplementary S1

3D imaging of tumor developed 2 weeks after dorsal wall targeted electroporation (DWE; A and A’) or medial wall targeted electroporation (MWE; B and B’). A and B present raw images and A’ and B’ show the segmented mCherry signal of tumors. (C) Graphs representing the growth of the tumor volume at 1, 2, 3 and 4 wpe in animals after DWE or MWE. Each circle represents one animal. The mean volume per time point per condition is drawn as a bar. Scale bars= 1000 µm.

Figure S2

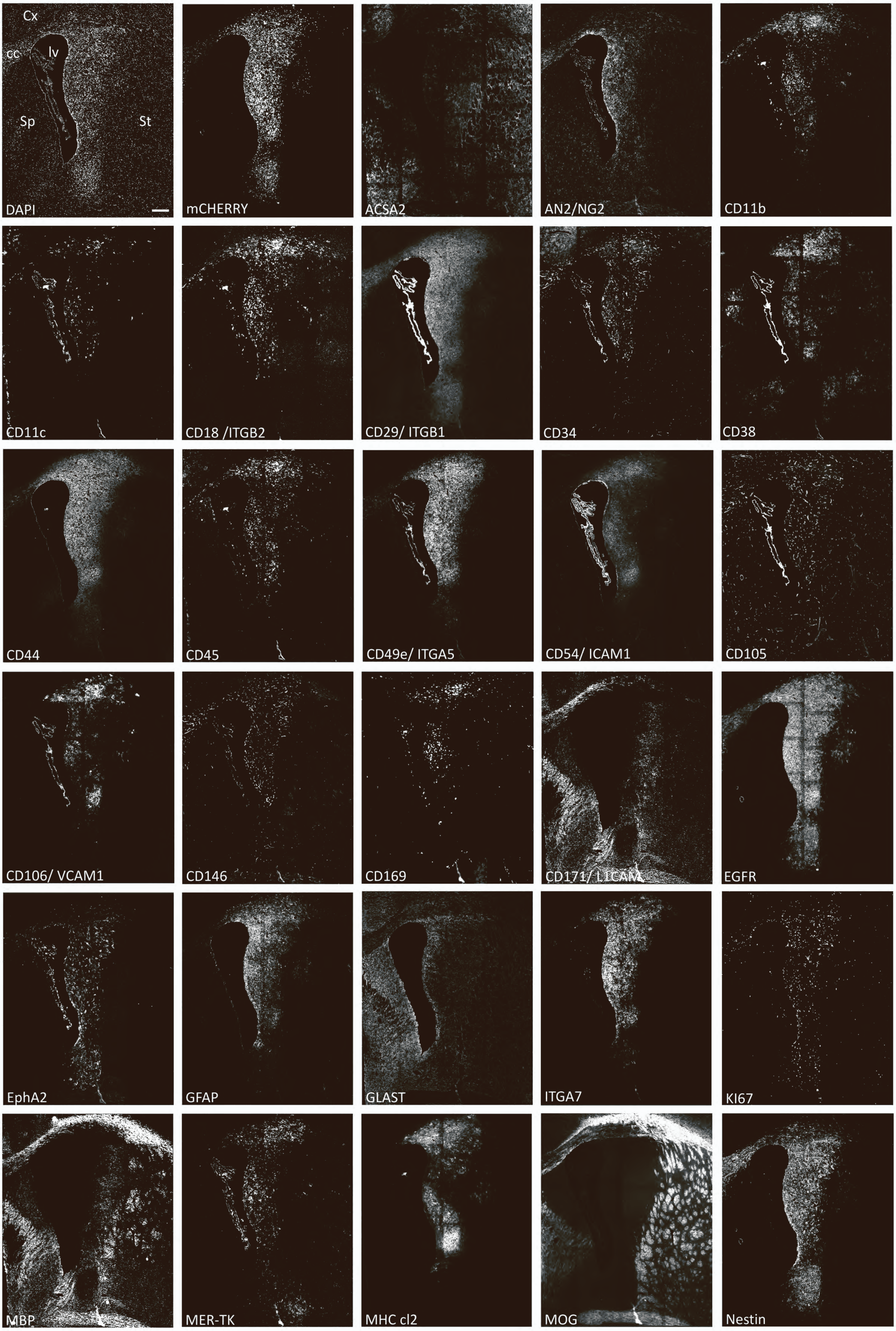
Cx
cc
lv
Sp
St
DAPI
mCHERRY
ACSA2
AN2/NG2
CD11b
CD11c
CD18 /ITGB2
CD29/ ITGB1
CD34
CD38
CD44
CD45
CD49e/ ITGA5
CD54/ ICAM1
CD105
CD106/ VCAM1
CD146
CD169
CD171/ L1CAM
EGFR
EphA2
GFAP
GLAST
ITGA7
KI67
MBP
MER-TK
MHC cl2
MOG
Nestin

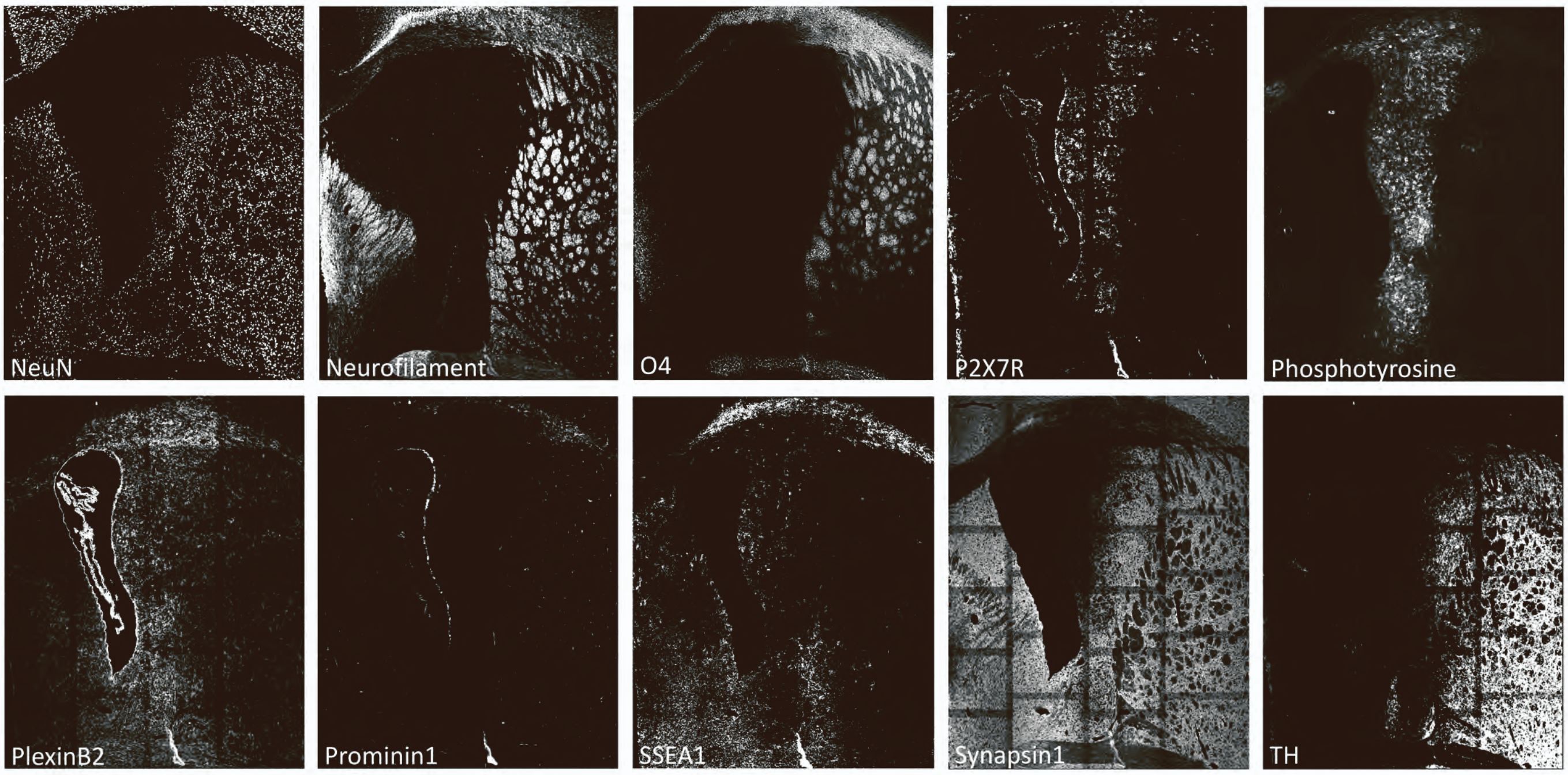

Supplementary S2

Single staining of DAPI, mCherry and each of the 38 selected antibodies used in MICS analyses on the same selected coronal section as shown in Figs 4 and 5 at 2wpe after LWE. Of note, for some of the markers we observed a tiling pattern, representing a bleaching artefact due to double exposure necessary for subsequent stitching. Wherever possible these lower stained regions were excluded from analyses.

Scale bars= 250 µm. cc: corpus callosum; Cx: cortex; Sp: septum; St: striatum.

Figure S3

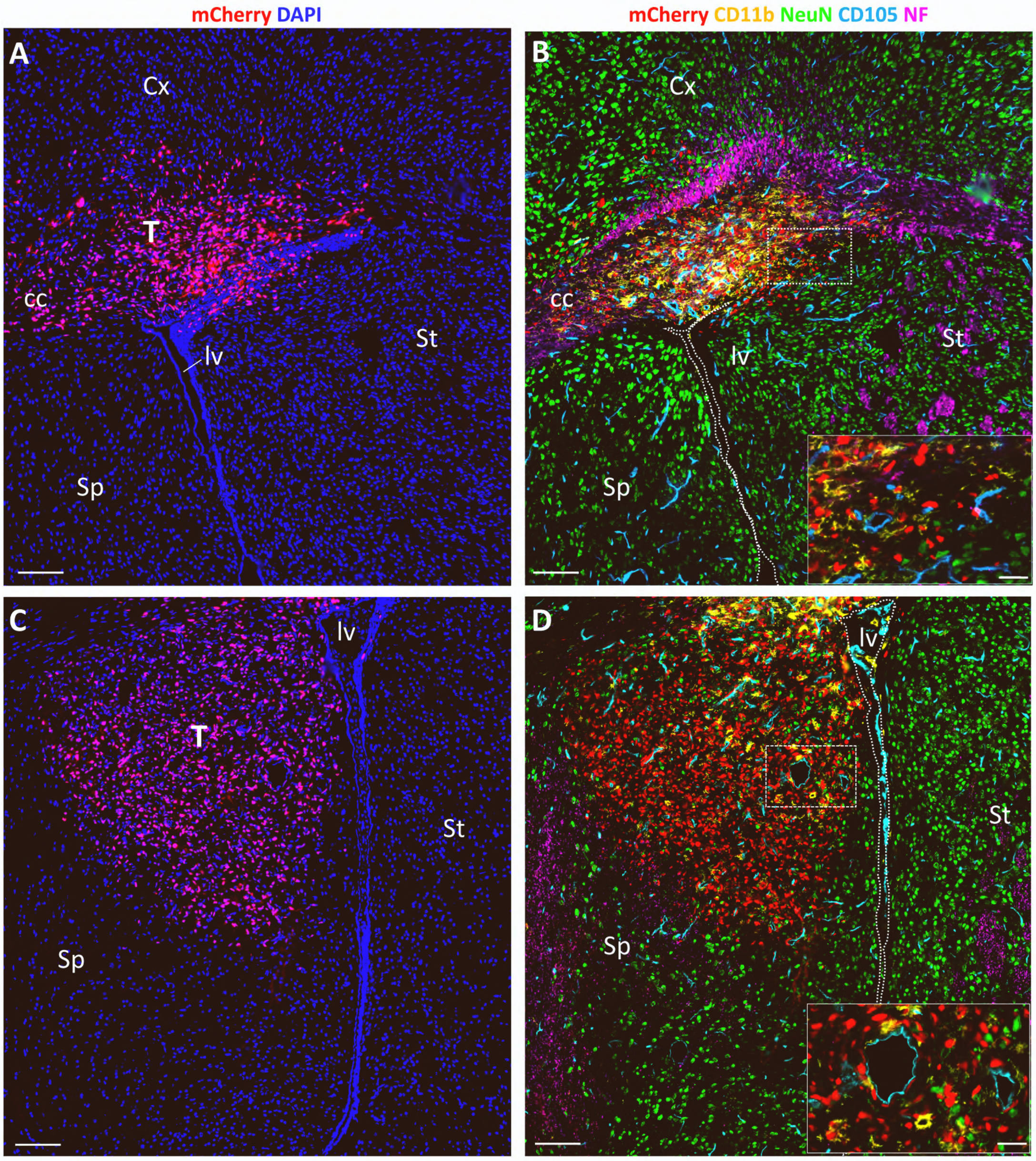

Supplementary S3

High resolution histological analyses using MICS.

(A and C) Coronal sections used for MICS analyses showing mCherry-positive cancer cells after dorsal wall electroporation (DWE; A) and medial wall electroporation (MWE; C).

(B and D) Ultrahigh content imaging of these DWE (B) and MWE (D) sections with markers for cancer cells (mCherry), myeloid cells (CD11b), vascular endothelial cells (CD105), neurons (NeuN) and axonal tracts (NF; neurofilament). The boxed areas delineate high magnifications presented in the lower right corner, allowing the identification of the different cell types at cellular resolution.

cc: corpus callosum; Cx: Cortex, lv: lateral ventricle, Sp: Septum; St: Striatum, T: Tumor. Scale bars= 500 µm (50 µm in insets)

Figure S4

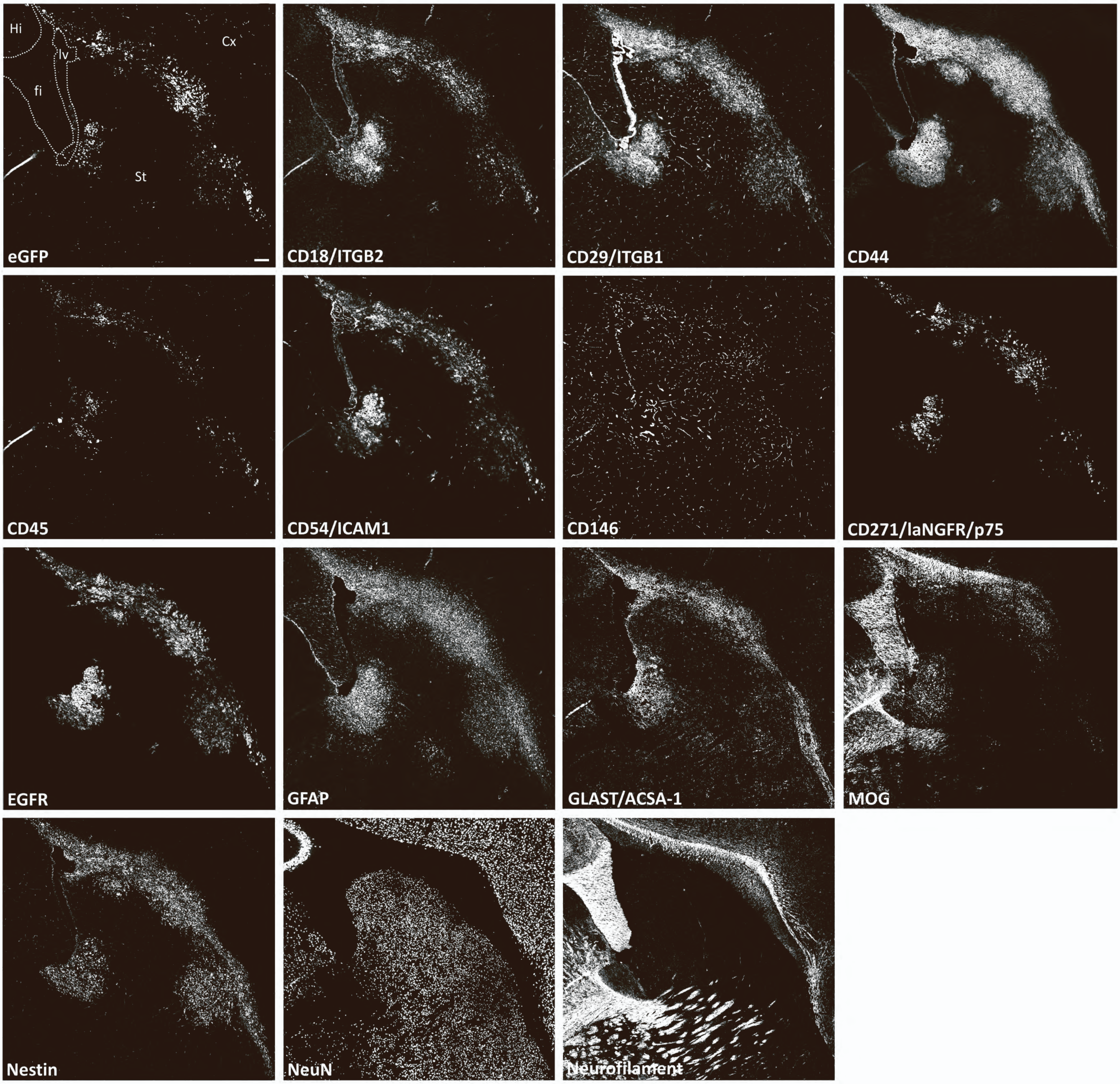

Supplementary S4

Individual staining patterns on the same tissue section as shown in Figs 6 and 7 of 14 antibodies functional in the 3D-2D approach as listed in Table 2 (plus eGFP labeling).

Scale bar= 100 µm. cc: corpus callosum, Cx: Cortex, fi: fimbria, Hi: Hippocampus, lv: lateral ventricle, St: Striatum.
